# Assessing Australia’s future solar power ramps with climate projections

**DOI:** 10.1038/s41598-023-38566-z

**Published:** 2023-08-02

**Authors:** Shukla Poddar, Jason P. Evans, Merlinde Kay, Abhnil Prasad, Stephen Bremner

**Affiliations:** 1 School of Photovoltaic and Renewable Energy Engineering, University of New South Wales, Sydney, Australia; 2 ARC Centre of Excellence for Climate Extremes, University of New South Wales, Sydney, Australia; 3 Climate Change Research Centre, Biological, Earth and Environmental Sciences, University of New South Wales, Sydney, Australia

**Keywords:** Climate-change impacts, Projection and prediction, Photovoltaics

## Abstract

Increasing levels of photovoltaic (PV) penetration to the electricity grid brings challenges to both design and operation of the grid due to its vulnerability to climate change. A crucial aspect of PV operation is power ramps leading to variability and instability in the grid. With notable large-scale PV deployment planned, including the world’s largest planned solar energy infrastructure in Powell Creek Australia, characterising future ramps is crucial for ensuring stable power generation to support large-scale economic development. Using CORDEX-Australasia projections under RCP8.5 and RCP4.5 emission scenarios, future solar ramps across Australia have been characterised up to 2100. Results predict a reduction in ramp magnitude across Australia, with changes in frequency and period length varying with the location. This work highlights the importance of considering future changes in climate when designing large-scale solar farms to ensure the incorporation of frequency control devices and storage plans for a reliable power supply.

## Introduction

The installed capacity of grid-connected solar power systems is rapidly increasing globally^1^. However, the integration of large-scale photovoltaic (PV) systems into the electricity grid poses a significant technical challenge due to the variable nature of the solar resource. Fluctuations in the global horizontal irradiance (GHI) caused by cloud movements are responsible for intermittent periods of PV power output. On a clear-sky day, the PV power generated is expected to follow a predictable diurnal curve similar to the GHI at that location^2^. However, this diurnal curve abruptly changes due to cloud movements that may result in a sudden increase or decrease of the output (called ramps). Scattered fair-weather cumulus clouds can generate ramps varying from seconds to minutes, while a deck of opaque stratus clouds can generate ramps that decrease energy output for several hours^3^. Thus, ramps affect the quantity of electricity generated and reliability of PV systems. At higher solar penetration levels, sudden fluctuations in the amount of PV power produced can adversely affect the operation of power systems and the supply–demand ratio across different timescales^4^. To meet the local electricity demand, the grid operators must respond to the cloud-induced PV electricity fluctuations and balance the significant surplus or deficit generation from the embedded PV generators. Ramps of a shorter duration (in seconds) can cause local voltage flicker, which increases the need for regulation equipment (e.g., on-load tap changers) and thus increases maintenance costs. At longer timescales (in minutes), variations in the power produced by PV panels can significantly impact grid stability and the power quality^5^. Hence it is essential to identify and predict ramp occurrence for planning storage solutions and technological developments in ramp control devices.

Solar ramps have been studied for different parts of the world^5–8^ using PV power output^2,9^ or GHI^5,10^ observations. These studies have quantified the ramp events at a PV plant scale and have highlighted their impact on the grid. Variability in power generated is affected by the sky conditions^5,11,12^ influenced by the local weather events^2,10,13^. Few studies have identified the localized weather events responsible for the occurrence of ramps^2,9,13^ and also studied their seasonal and annual variability^9^. The future changes in the cloud cover conditions and weather patterns due to climate change will influence the occurrence of ramps in different parts of the world.

Despite several studies on solar ramps, most are based on observations spanning less than two years. Furthermore, the previous research in this field is either inclined towards developing new forecasting techniques^14–17^ or identifying the ramp behaviours over site-specific large-scale PV plants using historical data^2,6,18^. Minimal research has been conducted on a larger spatial scale to examine the ramping distribution patterns. No study has been undertaken to suggest how solar power ramp properties will change due to climate change. Australia has one of the world's best solar resources, and there has been a rapid increase in deployment of both large-scale and small-scale PV across Australia to meet the net zero targets^19^. With the increase in demand for solar electricity generation and integration in Australia, it is essential to understand the nature and magnitude of such variations in PV power at different timescales to plan storage solutions and stable grid regulation. Even though few studies related to GHI variability over Australia have been undertaken^4,20^ in the past, limited studies have focused on solar power ramps over Australia^21,22^, with no studies related to ramp events Australia-wide to date.

The main goal of this study is to quantify the overall spatio-temporal picture of ramp distribution over Australia and estimate future changes in ramps under climate change scenarios. In this study, we use the PVLIB python package to obtain PV power output and intercompare the solar power ramps for different timescales to test the applicability of 1-hour data to assess future ramps. We identify the ramp events from the regional climate model (RCM) simulations from the Coordinated Regional Downscaling Experiment (CORDEX) for Australasia^23^ for the historical (1976–2005) and future period (2070–2099: far future) under an intermediate and high-emission scenario (representative concentration pathway (RCP) 4.5 and RCP8.5). We estimate the future changes in ramp magnitude, frequency and periods due to climate change over Australia for the first time. This analysis will be beneficial to policymakers and stakeholders in informing site selection and planning storage solutions to overcome future intermittency issues. Further, the research work presented will provide a reference to estimate future solar ramp events spatiotemporally and can be applied across different parts of the world.

## Results

### Future changes in ramp characteristics across Australia

On comparing, the ramp characteristics of the 5-minute and hourly dataset for monocrystalline silicon (Mono-Si), multi-crystalline silicon (Mc-Si) and thin film cadmium-telluride (CdTe) PV technologies, the ramp distribution show broad agreement with each other. Ramps detected with hourly data overestimate the frequency of low-magnitude ramps and underestimate the frequency of high magnitude ramps compared to 5-minute data. The ramp characteristics for the three technologies were found to be similar. The thermal properties of different PV technologies do not have any discernible effect on ramps (refer to methods). The following sections describe ramp characteristics using CORDEX-Australasia hourly data for the historical and far future period only for Mono-Si since it is a dominant PV technology, and the ramp behavior is similar for different PV technologies. The results for the near future period are included in the supplementary material.

### Ramp magnitude

**Figure 1:**
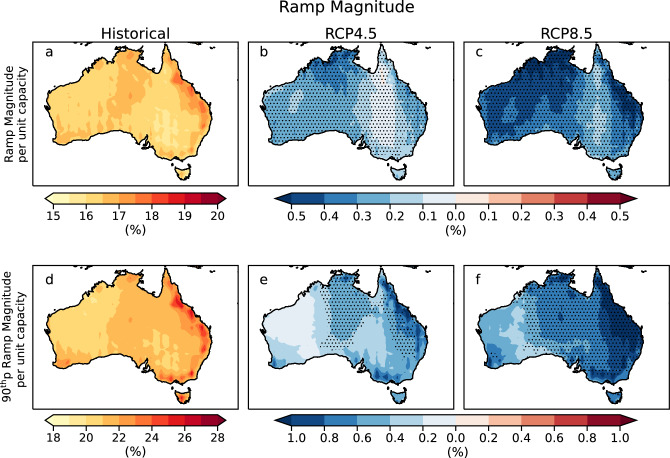
Ramp magnitude across Australia. Panel (**a**) represents the mean ramp magnitude during the historical period (1976–2005). Panel (**b**) and (**c**) represent the future changes in mean ramp magnitude for the far future (2070–2099) period under RCP4.5 and RCP8.5. Panel (**d**) represents the ramp magnitude at the 90th percentile during the historical period (1976–2005). Panel (**e**) and (**f**) represent the future changes in ramp magnitude at the 90th percentile for the far future (2070–2099) under RCP4.5 and RCP8.5. Stippling indicates a significant change (according to methods: significance test).

The magnitude of solar power ramps depends on the abruptness of changes in solar radiation due to cloud movements. The mean ramp magnitude is highest for Eastern coastal Australia (> 17.5% of the installed capacity) during the historical period (Fig. 1a). The ramp magnitude is projected to significantly decline ~ 0.4 to 0.5% under RCP4.5 (Fig. 1b) and > 0.45% under the RCP8.5 scenario (Fig. 1c) in the far future. Northern Australia is expected to have the highest decline in the ramp magnitude for RCP4.5. Under the RCP8.5 scenario, we expect a maximum decline in Australia's Northern and Eastern regions. We analyze the ramps at the 90th percentile to assess the extreme ramp events. The ramp magnitude at the 90th percentile (referred to as extreme ramps here) is highest near the east coast of Australia during the historical period (Fig. 1d). These extreme ramps are projected to decline throughout the country in the future for both periods under RCP4.5 and RCP8.5 emission scenarios (Fig. 1e,f). During the far future period, the magnitude decreases further under both scenarios. The highest decline occurs in the East and some parts of North Australia (up to 1.5% of the installed capacity). Additionally, it is interesting to note that even though the maximum decline in the mean ramp magnitude is in the Western part of the continent in the future, the maximum reduction in extreme ramp magnitude is projected in the East and North. This reveals that cloud-induced variability in PV generation for future periods is projected to decrease; hence, the requirement for an extensive storage facility to maintain grid stability at all times of the day will reduce.

Additionally, the impact of ramps greatly depends on the region’s electricity demand. The power quality and grid stability become a critical concern during high electricity demand accompanied with variable energy generation^24,25^. In Australia, New South Wales and Queensland have the highest electricity demand. According to Australian Energy Market Operator, the overall electricity demand is projected to increase by 47.5% and 50% respectively in New South Wales and Queensland by 2050^26^. Our results project highest reduction in extreme ramp magnitude in these regions. This will be beneficial for maintaining the supply–demand ratio, thus ensuring grid-stability. Variable power generation can induce voltage fluctuations and cause a deviation from the nominal operating frequency, thereby reducing the quality of the power and creating instability in the grid. If the distribution system is unable to compensate for the decrease in power output, it may lead to voltage collapse and subsequent power outages^24^. Moreover, reduction in ramp magnitude indicates less extensive storage requirements, which can be economically beneficial to the grid-connected large-scale solar plants.

### Ramp frequency

**Figure 2:**
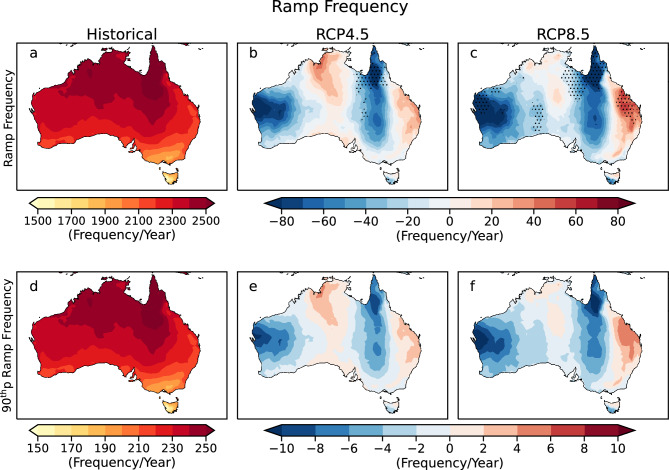
Ramp frequency across Australia. Panel (**a**) represents the mean ramp frequency during the historical period (1976–2005). Panel (**b**) and (**c**) represent the future changes in mean ramp frequency per year for the far future (2070–2099) period under RCP4.5 and RCP8.5. Panel (**d**) represents the frequency of ramps with ramp magnitude at the 90th percentile during the historical period (1976–2005). Panel (**e**) and (**f**) represent the future changes in the frequency of ramps with ramp magnitude at the 90th percentile for the far future (2070–2099) period under RCP4.5 and RCP8.5. Stippling indicates a significant change (according to methods: significance test).

The highest number of ramp events occur in the North during the historical period (~ 2400 to 2500 per year; Fig. 2a). During the near future period, the ramp frequency decreases for most of the country under RCP4.5 (supplementary Fig. 9s). A higher significant decline in ramp event occurrence is predicted in the West and Northern Queensland (~ 60 per year) under RCP8.5 (supplementary Fig. 9s) in the near future. Further, it can be noted that the spatial pattern of these changes remains the same under both scenarios for the far future period as that of the near future; however, the magnitude intensifies for the far future period (Fig. 2b,c). It is projected that the ramp events increase in Northern and Eastern Australia under RCP4.5, unlike RCP8.5, where the increases are confined to East Australia.

On analyzing the extreme ramps, we find Northern Australia is prone to more frequent extreme ramps during the historical period (> 200 per year) (Fig. 2d). It is predicted that there will be a slight increase in the number of extreme ramps in some parts of North and East Australia in the future, with decreases elsewhere. The maximum increases in the extreme ramps are predicted in the North and East of the continent (up to 4 per year) during the far future period under RCP4.5 (Fig. 2e), while similar increases are expected to occur only near the East (up to 6 per year) under RCP8.5 (Fig. 2f). Further, it can be noted that the spatial patterns for the changes in mean ramp frequency are similar to the changes in the extreme ramp frequency. This indicates that the peak and the tail of ramp frequency distribution will shift in a similar direction in the future. The future reduction in the number of ramp events at a location indicates that fewer onload tap-change operations will be required to maintain constant voltage, thus reducing the chances of grid imbalance and reducing the installation and maintenance costs of ramp control devices in the future. However, regions with future increases in ramp frequency require more robust ramp control devices to avoid grid instability and voltage flicker issues.

### Ramping period duration

**Figure 3:**
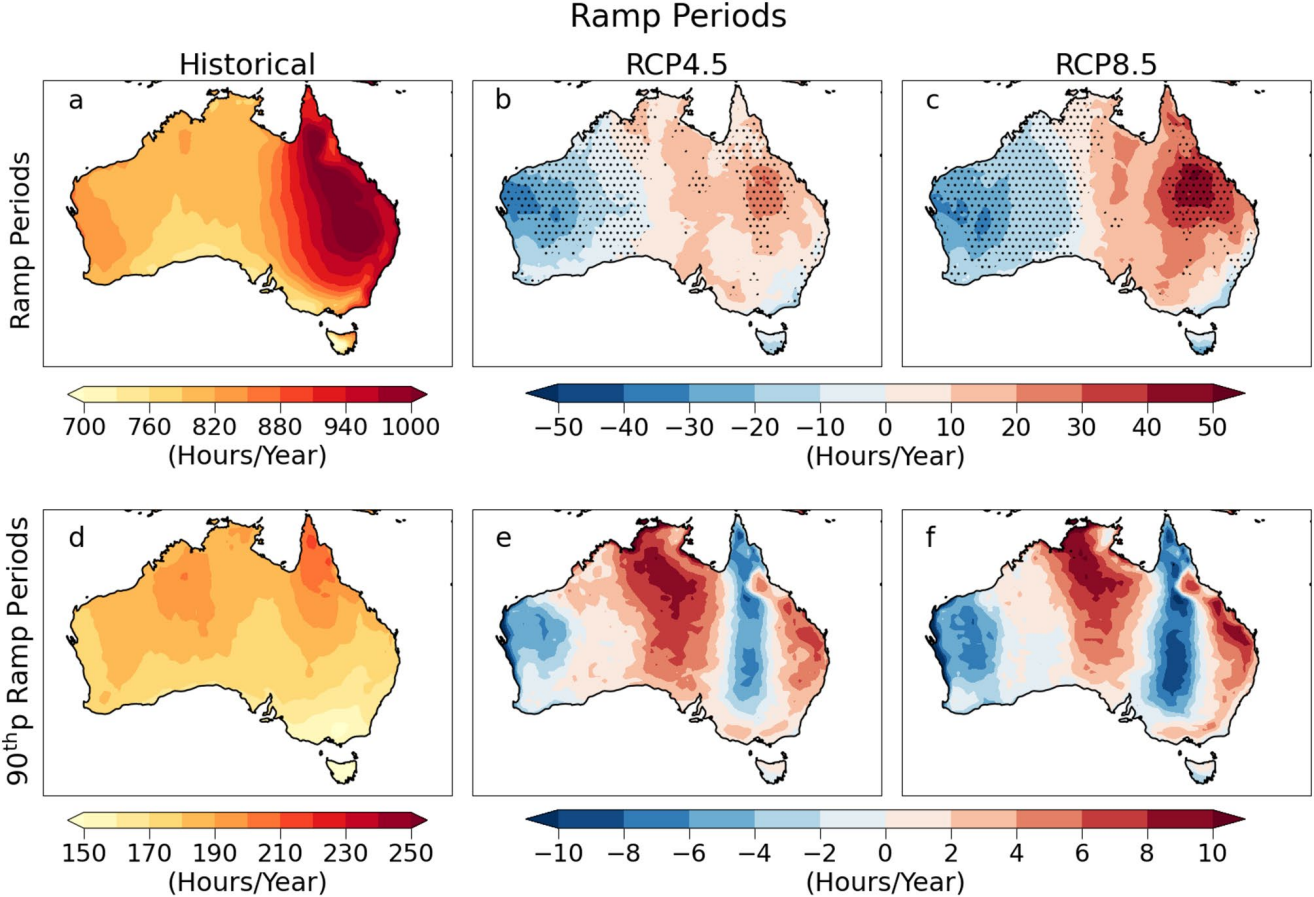
Ramping periods across Australia. Panel (**a**) represents the mean ramp periods during the historical period (1976–2005). Panel (**b**) and (**c**) represent the future changes in mean ramp periods per year for the far future (2070–2099) period under RCP4.5 and RCP8.5. Panel (**d**) represents the ramp periods with ramp magnitude at the 90th percentile during the historical period (1976–2005). Panel (**e**) and (**f**) represent the future changes in ramp periods with ramp magnitude at the 90th percentile for the far future (2070–2099) under RCP4.5 and RCP8.5. Stippling indicates a significant change (according to methods: significance test).

In this study, the time interval over which ramp events are detected is termed ramping period duration. The identified ramp event might be detected only for a single time interval or ramps may occur over consecutive periods in a day. This is produced by scattered or variable cloud cover passing over the site. Hence, the ramping period duration can vary from 60 minutes onwards for an hourly dataset. The highest mean ramping periods occur over Eastern Australia (> 150 min/day) during the historical period (Fig. 3a). During the far future period, the ramping periods are projected to decrease in the West and increase in the East under both scenarios (Fig. 3b,c). There is a significant reduction in ramping periods near the West and South-Eastern Coast under both scenarios in the far future period. During the historical period, Northern Australia experienced the most extended periods of extreme ramps (Fig. 3d). It is interesting to note that the maximum increases in future extreme ramp periods are near Northern, Central and Eastern coastal regions of Australia for both scenarios (Fig. 3e,f), unlike the mean ramp periods with increases confined to Eastern Australia.

Locations with a decline in ramp frequency, ramp periods and ramp magnitude (like Western Australia) suggest the possibility of fewer and lower magnitude ramps with shorter duration. These regions are projected to have lower cloud-induced PV power variability in the future; thus, a more reliable power supply and are highly stable for future expansion of PV technology in the electricity grid. However, regions like East Australia, with a projected increase in ramp frequency and ramp periods, suggest the possibility of more frequent lower-intensity ramps for prolonged periods in the future. Regions with a decline in ramp magnitude and frequency but increases in ramp periods might face the possibility of prolonged lower magnitude ramps with reduced chances of occurrence. This would likely create extended periods of energy deficits in these regions and would require dependency on storage systems to curb the supply–demand imbalance.

### Case study: Powell creek solar farm

**Figure 4:**
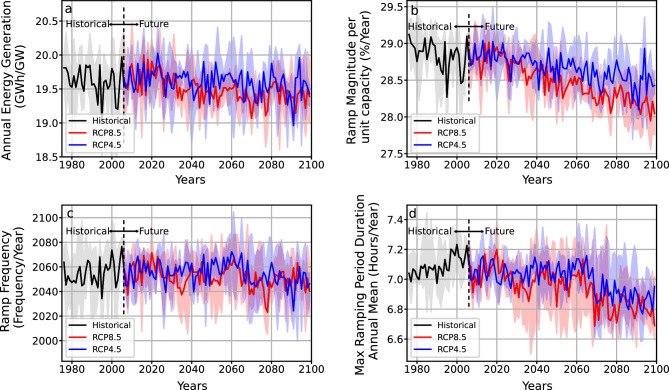
Time series of annual power generation, ramp magnitude per unit capacity, ramp frequency and maximum annual mean ramping period duration per year for Sun Cable’s Powell Creek solar farm located in the Northern Territory, Australia. The black line represents the ensemble mean for the historical period. The blue and red lines represent the ensemble mean for the future periods under RCP4.5 and RCP8.5, respectively. The shading in the grey, blue and red represents the interquartile range of the ensemble members for the historical and future.

Sun Cable’s Powell Creek solar farm is the largest solar farm in the world under the developmental phase. It is to be located in the Northern Territory, Australia, with 20GW of proposed solar generation capacity and a 5000 km transmission system to supply Darwin and Singapore electricity generated from reliable renewable energy sources. We demonstrate the future changes in the power generation capacity and ramp behavior for the Powell Creek solar farm to estimate the storage requirements and the need for extensive planning of ramp control. The time series representing Mono-Si technology's annual power generation capacity, ramp magnitude per year, ramp frequency per year and maximum ramping period duration per year are shown in Fig. 4a–d. The PV technologies and their specifications to be used in the Powell Creek solar farm is yet to be decided as the solar farm is still in its planning and developmental phase. Thus, we have calculated the ramps considering the mono-Si module specifications mentioned in the supplementary Table 1s. The ensemble spread is comprehensive for all variables (blue and red shading). This indicates that different modelled futures can produce different PV power futures, and one should be cautious of results produced using a single climate model.

We estimate normalized power generation using the mono-Si module specifications in Table 1s and scale it as per the proposed generation capacity (20GW) for Powell Creek to understand the future changes in the power generation at the plant. It can be noted that there is a decline in the annual generation capacity of the farm (up to 1GWh/GW) in the future under both scenarios (Fig. 4a). Our results indicate ~ 5% decrease in energy generation from the plant due to climate change. This estimation does not consider any future solar farm production capacity expansion.

It is predicted that the mean ramp magnitude will decrease (up to 1% under RCP8.5 and 0.5% under RCP4.5) by the end of the century. Further, the frequency of ramp event occurrences is expected to increase till 2060 and decrease by the end of the century with noticeable decadal variability. However, we expect a slight increase in the ramping periods during the near future, followed by a steep decline until the end of the century. This indicates the possibility of lower-magnitude ramp occurrences in the future with shorter durations. Thus, while there will be persistent cloud-induced intermittency issues in the future, these will decrease, suggesting that current ramp-control devices will remain suitable under future climates. The results highlight the importance of establishing an appropriate storage facility to manage curtailment and energy deficit periods to maintain a supply–demand ratio.

## Discussion

Weather-induced variability in solar power generation at shorter timescales induces stress on the electricity generators to maintain a stable supply–demand ratio. Sudden solar power fluctuations, called ramps, and their associated characteristics must be accurately characterized to enhance ramp forecast accuracy and reduce grid instability. Estimating solar power ramp capacity and frequency is essential for implementing proper storage facilities at the plant to overcome intermittency issues. This paper assesses the short-term power fluctuations and examines projections of cloud-induced solar power ramps in Australia under different future climate scenarios. The expected future changes in ramp features are studied using the dynamically downscaled regional climate model data from CORDEX-Australasia simulations under an intermediate and high-emission scenario. Our results predict up to 5% changes in the future ramp frequency and periods when compared to the historical baseline. We observe that even though the impact of climate change on future changes in ramp magnitude is statistically significant almost everywhere, the changes are less than 1.5% of the installed capacity. The regions showing statistically significant future changes in these characteristics are denoted in stippling.

The mean ramp capacity is expected to decline throughout the continent by the end of the century under both RCP4.5 (up to 0.5%) and RCP8.5 (up to 1%) emission scenarios. We find that the magnitude of these changes is higher for RCP8.5 with the highest decline near the North and parts of Northeast Australia unlike under RCP4.5, where the maximum decline in mean ramp capacity is confined to the North. Under both RCP8.5 and RCP4.5 scenarios, the mean ramp frequency is projected to increase near the East coast and parts of Northern Australia (up to 60 per year), with decreases elsewhere (> 80 per year) by the end of the century. The mean ramping period duration is expected to significantly increase, with the increase mostly observed in Northern and Eastern Queensland under both scenarios (up to 50 h per year) by the end of the century. These changes reach up to 5% of the historical values are statistically significant near Queensland and the western and south-western regions of Australia. These are the regions where the future changes are large compared to the variability in the ensemble members. The future changes in ramp frequency and periods are higher under the RCP8.5 scenario. It is important to emphasize that the results show the dependence of ramps on future emission scenarios. Our results highlight that different emission scenarios used by regional climate models can significantly affect the magnitude of future changes in ramp characteristics. The high emission scenario RCP8.5 projects up to two times higher future changes in ramp characteristics than the intermediate emission scenario RCP4.5. Hence, it is essential to estimate the future intermittency by considering different emission scenarios to accurately assess the storage requirements for reliable and stable grid operation in the future.

This paper examines the outcomes of the CORDEX-Australasia ensemble mean. However, it is important to acknowledge that each member of the ensemble possesses unique characteristics in terms of future projections due to variations in parameterization schemes and driving general climate models (GCMs). This results in a range of potential future prospects. Therefore, we have comprehensively analyzed ramp characteristics derived from each RCM-GCM combination, which is presented in the supplementary Figs. 11s–22s. The findings demonstrate that while most ensemble members exhibit similar future changes, a few members indicate the possibility of experiencing opposite changes. This underscores the significance of utilizing multiple ensemble members to assess the level of confidence in future projections accurately. This work uses six ensemble members that had future projections for both RCP8.5 and RCP4.5, it is recommended to expand the size of the RCM ensemble in future studies to better sample the future climate change and increase the confidence in the results obtained.

PV systems are expected to expand broadly throughout Australia over the twenty-first century with several technological advancements. With increased penetration levels of PV systems into the electricity grid, it is challenging to tackle weather-induced variability at all timescales to manage voltage fluctuations. Understanding future ramp events under different climate scenarios is essential for several technological advancements in ramp control devices and intensive research on grid operations like power generation, storage, transmission, and distribution. Further, it is of utmost importance to consider the future changes in the intermittency and ramp features due to climate change while selecting optimal locations for deploying future PV plants. In this regard, this work lays the foundation for such analysis, and it is expected that future work will explore the implication of the findings presented here. Further work exploring the seasonality of ramps across Australia and the synoptic features leading to ramp events are highly recommended. The proposed framework to study the future ramp events spatiotemporally can be further extended across different parts of the world using climate model data during the planning, developmental and construction phase of the solar farms to ensure optimum grid operation and reduce redundant power supply.

## Data and methods

### Data

In this study, we use meteorological observational data (temperature, GHI, wind speed, relative humidity, plane of array (POA) irradiance) and PV power data from Desert Knowledge Australia Solar Centre (DKASC), Alice Springs solar farm for the period 2010- 2016 recorded at 5-minute intervals. The DKASC has several solar technologies (Mono-Si, Mc-Si, CdTe cells) with fixed, single and dual-axis tracking systems. Supplementary Table 1s includes the PV technologies and their configurations for simulating power in PVLIB.

We use regional climate model simulations from CORDEX-Australasia^23^ to study the impact of climate change on future ramps. Two RCMs were used to downscale three global climate models (GCMs) from the Coupled Model Intercomparison Project 5 (CMIP5) to obtain the climate projections over the CORDEX-Australasia region, performed as part of the NSW / ACT Regional Climate Modelling (NARCliM)1.5 project^27^, have been used in this study. The climate projections obtained using 2 RCMs and 3 GCMs pair create a 6-member ensemble for historical and future periods (supplementary Table 6s). These RCM-GCM pairs were selected for the study as they provided the highest temporal resolution (1-hourly) data for historical and future scenarios. The future simulations are obtained for the RCP4.5 and RCP8.5 scenarios. The periods 1976–2005, 2030–2059 and 2070–2099 are analyzed as historical, near future and far future periods.

CORDEX-Australasia ensembles have been evaluated for the historical period and are found to reproduce many aspects of the climate of the region like minimum temperature, maximum temperature and precipitation^23,28^. The CORDEX-Australasia shortwave radiation has been evaluated using the reanalysis datasets and is found to capture the spatial pattern and magnitude with reasonable fidelity (supplementary Fig. 1s). Further, the future changes in the land-use type and enlargement of arid areas were also studied using CORDEX projections^29^. In this study, we have used 1-hourly shortwave downward radiation, temperature, wind speed, relative humidity and pressure to obtain the power projections for different PV technologies.

## Methods

### PV power simulations using PVLIB

PVLIB is an open-source PV power modelling tool developed at Sandia National Laboratory^30^. This study uses the PVLIB python package to model the ground-mounted PV system. The detailed configuration of the PV technologies to simulate power is listed in supplementary Table 1s. This approach identifies the following main modelling steps: separation of the GHI into the beam and diffuse components; transposition of the horizontal irradiance to the tilted plane of the PV array; calculation of the clear sky radiation components and cell temperature; and the DC/AC power output (supplementary Fig. 2s). These steps involve the calculation of the position of the Sun. The general inputs required for modelling power are GHI, temperature and wind speed. The various models chosen for the PV power modelling are listed in Table 1. The best-performing models with the least error on validation have been selected for decomposition, transposition and clear sky modelling (refer to supplementary section 3). The temperature model, DC and AC model have been chosen from previous literature due to a lack of observations for validation purposes^31–33^. Initially, meteorological data from DKASC is used to simulate the power and validated with the power output of the PV technologies. Later, similar modelling steps are followed to simulate power output using hourly CORDEX-Australasia projections.

**Table 1:** Details of the various models used for simulating power using PVLIB.

	Model name	Input parameters	References
Decomposition model	DISC	GHI, solar zenith angles, pressure, airmass	^34^
Transposition model	Isotropic	Surface tilt, diffuse horizontal irradiance	^35^
Clear sky model	sSOLIS	apparent elevation, aod700, precipitable water, extra-terrestrial irradiance, pressure	^36^
Temperature model	SAPM	POA GHI, wind speed, temperature, module parameters, reference irradiation	^31^
DC model	SAPM	Irradiance reaching module, cell temperature, module parameters	^31^
AC model	PV watts inverter	Irradiance transmitted to PV cells, cell temperature, dc power at 1000 W/m^2^, temperature coefficient of power, reference cell temperature	^32^

### Identification of ramps

Ramps correspond to sudden localized changes in a power time series. The ramps have been obtained for successive points in the time series using the method proposed in the recent report by the Australian Energy Market Operator^37^:

$${\text{R}}({\text{t}}) = \left| {{\text{P}}({\text{t}}) - {\text{P}}({\text{t}} - \Delta {\text{t}})} \right|$$

where P is the power at time step t, △t is the time interval. Following the report^37^, only the step changes in the power series are considered significant ramps if the absolute value exceeds 10% capacity in the time interval. We perform the ramp analysis for different temporal resolutions (corresponding to 5 minute and 1-hourly) to understand their distribution patterns and magnitude. While some studies use observational datasets having temporal resolutions of 5-minutes^11,12^ or better ^13,22,38^, others use half-hourly or larger^11,39^ time steps. Model projections till the end of the century are computationally expensive and are generally available at hourly timesteps. Here we examine the representation of ramps at both temporal resolutions (5-minute and hourly) to understand whether hourly data is adequate to characterise ramp behaviour. All the ramp analyses are performed by considering the absolute changes in power variability and indicating absolute ramp characteristics. Absolute ramp magnitude, frequency and periods are termed ramp magnitude, frequency and periods in the rest of the paper.

### Significance test

We use the student’s *t*-test at a 5% significance level (*p* < 0.05) to examine the statistical significance of the results and present it based on the convention of Tebaldi et al^40^. Student’s *t*-test is performed at each grid point, assuming equal variances for the past and future periods to test the significance of the mean change in each ensemble member. Significant change is shown with stippling. These are the grid points where at least four ensemble members show a significant change and also agree on the direction of change. These regions have high confidence in future change.

### Ramp characteristics at different timescales

**Figure 5:**
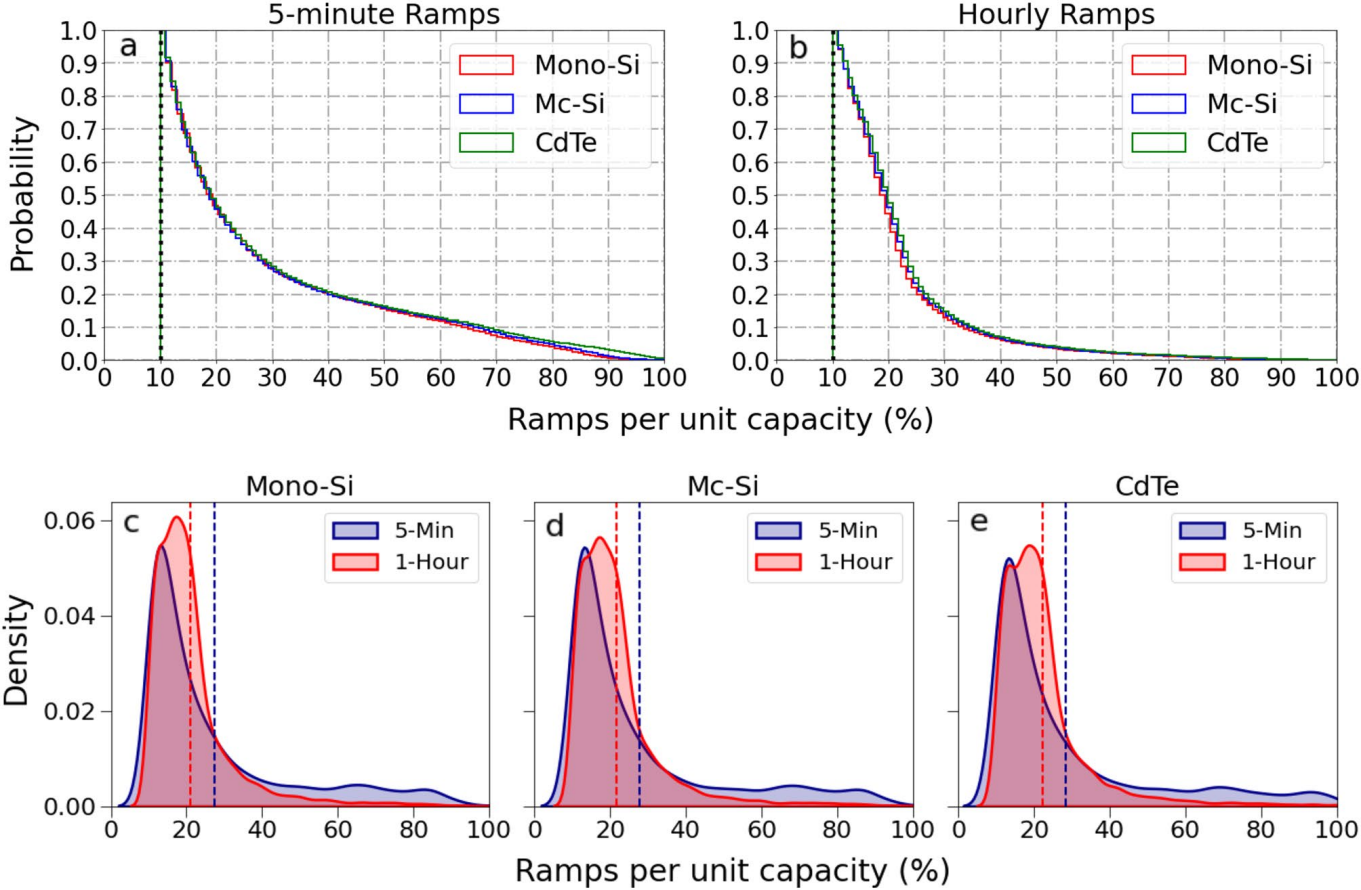
Cumulative probability distribution and density distribution plots of ramp magnitude per unit capacity mono-crystalline Silicon (Mono-Si), multi-crystalline silicon (Mc-Si) and thin film cadmium-telluride (CdTe) PV technology. (**a**) Cumulative probability distribution plot for 5-minute data and (**b**) 1 hourly data for several PV technologies. The y-axis of the figure depicts the inverse cumulative density function for easy readability. Density distribution plots of ramp magnitude per unit capacity for (**c**) Mono-Si, (**d**) Mc-Si and (**e**) CdTe PV technology for 5-minute and hourly ramps. The Blue and red dashed line represents the average ramp magnitude per unit capacity for 5-minute and hourly ramps, respectively.

Ramp behaviour and characteristics depend highly on the temporal timescales at which they are calculated. We use regional climate projections with hourly output to investigate future changes in ramp behaviour due to climate change. It is possible to gain a holistic understanding of the intrinsic relationship between ramp characteristics at different timescales by analyzing the effect of timestep variations on its magnitude and frequency. We compare the ramp magnitude (represented in terms of per unit capacity) and frequency at 5-minute and 1-hourly timescales to assess their similarities and differences (Fig. 5). The results indicate that the magnitude of the ramps decreases with the increase in the time interval. The cumulative distribution plots of ramp per unit capacity at 5-minute and hourly timescales (Fig. 5a,b) show that it tails off at 60% for hourly timescales but around 80% for 5-minute timescales with similar distributions for Mono-Si, Mc-Si and CdTe PV technologies. Further, the average ramp magnitude per unit capacity is similar for different PV technologies for both 5-minute and 1-hourly ramps (Table 2; Fig. 5c–e).

**Table 2:** Statistical analysis for 5-minute and hourly ramps per unit capacity.

	Mono-crystalline Silicon		Multi-crystalline Silicon		CdTe	
	5-min	1-h	5-min	1-h	5-min	1-h
Mean	27.31	21.14	27.62	21.69	28.39	22.23
P50	18.38	18.46	18.30	18.93	18.69	19.49
P90	63.64	32.92	65.92	33.91	67.72	34.72
Skewness	1.55	2.73	1.57	2.69	1.61	2.75

The table describes the mean, 50th percentile (P50), 90th percentile (P90) and skewness of 5-minute and hourly ramps for different PV technologies. The mean, P50 and P90 are expressed in %, while skewness has no units.

The density distribution plots show that the 5-minute ramps have lower peaks with heavier tails when compared to hourly ramps. This indicates that the frequency of occurrence of higher magnitude ramps is more common for ramps observed at a 5-minute timescale than hourly timescale. Hourly ramps have a more pronounced peak with thinner tails indicating the occurrence of more frequent smaller magnitude ramps. The distribution also displays a positive skewness for both 5-minute and hourly ramps. Overall, the hourly ramp distributions show a broad agreement with the 5-minute ramp distributions, noting that hourly ramps tend to overestimate the low-magnitude ramps and underestimate the higher-magnitude ramps slightly. Further, analyzing the frequency of ramp event occurrences in a 5-minute and 1-hourly dataset, it was found that 5-minute ramps occur ~ 5 times more than 1-hourly ramps. This analysis reveals relatively small differences in the ramp distribution observed at 5-minute and 1-hourly timescales and highlights hourly data's usefulness in studying ramp behavior when higher temporal resolution data is unavailable.

## Supplementary Information

Supplementary Information.

## Data Availability

No new data was generated in the study. The CORDEX-Australasia data used for the analysis are available from the Earth System Grid Federation (https://esgf-data.dkrz.de/search/cordex-dkrz/). ERA5 data were obtained from the ECMWF Climate Data Store (CDS), and MERRA-2 data were provided by NASA Goddard Earth Sciences (GES) Data and Information Services Center (DISC). The DKASC, Alice Springs data was available from https://dkasolarcentre.com.au/locations/alice-springs.
