# Supplementary material for: Assessing Australia’s future solar power ramps with climate projections
Source: Sci Rep. 2023 Aug 2;13:11503. doi: 10.1038/s41598-023-38566-z (PMC10397292; doi:10.1038/s41598-023-38566-z)
Supplement: Supplementary file 1 — Supplementary Information. [file 41598_2023_38566_MOESM1_ESM.pdf]

Supplementary Information for

Assessing Australia's future solar power ramps with climate projections

Shukla Poddar[1,2], Jason P Evans[2,3], Merlinde Kay[1], Abhnil Prasad[1,2,3], Stephen Bremner[1]

[1]School of Photovoltaic and Renewable Energy Engineering, University of New South Wales, Sydney, Australia

[2]ARC Centre of Excellence for Climate Extremes, University of New South Wales, Sydney, Australia

[3] Climate Change Research Centre, Biological, Earth and Environmental Sciences, University of New South Wales, Sydney, Australia

Corresponding author: Shukla Poddar (s.poddar@unsw.edu.au)

Contents of this file:

1. Table 1s-6s

2. Figures 1s to 22s

Introduction

This supporting information includes:

- Validation of CORDEX-Australasia ensemble mean radiation with two reanalysis products: MERRA2 and ERA5 (**Figure 1s**).
- Details of PV technologies and modelling steps for simulating power with PVLIB (**Figure 2s, Table 1s**).
- Evaluation of PVLIB model selection parameters (**Figure 3s, Table 2s-4s**).
- Validation of PVLIB power simulations with observations from DKASC solar farm (**Figure 4s, Table 5s**).
- CORDEX-Australasia RCM-GCM pairs used in the study (**Table 6s**).
- Mean power projections for from mono-crystalline silicon cells and future changes for various ensemble members under RCP4.5 and RCP8.5 scenario (**Figure 5s-7s**).
- Ramp characteristics for the near future period (**Figure 8s-10s**).
- Future changes in mean solar ramp magnitude across Australia for the near future and far future periods under RCP8.5 and RCP 4.5 scenario for the various ensemble members of CORDEX-Australasia (**Figure 11s-12s**).
- Future changes in extreme solar ramp magnitude across Australia for the near future and far future periods under RCP8.5 and RCP 4.5 scenario for the various ensemble members of CORDEX-Australasia (**Figure 13s-14s**).
- Future changes in mean solar ramp frequency across Australia for the near future and far future periods under RCP8.5 and RCP 4.5 scenario for the various ensemble members of CORDEX-Australasia (**Figure 15s-16s**).
- Future changes in extreme solar ramp frequency across Australia for the near future and far future periods under RCP8.5 and RCP 4.5 scenario for the various ensemble members of CORDEX-Australasia (**Figure 17s-18s**).
- Future changes in mean solar ramping periods across Australia for the near future and far future periods under RCP8.5 and RCP 4.5 scenario for the various ensemble members of CORDEX-Australasia (**Figure 19s-20s**).
- Future changes in extreme solar ramp duration across Australia for the near future and far future periods under RCP8.5 and RCP 4.5 scenario for the various ensemble members of CORDEX-Australasia (**Figure 21s-22s**).

1. **Validation of solar radiation from CORDEX-Australasia with reanalysis**

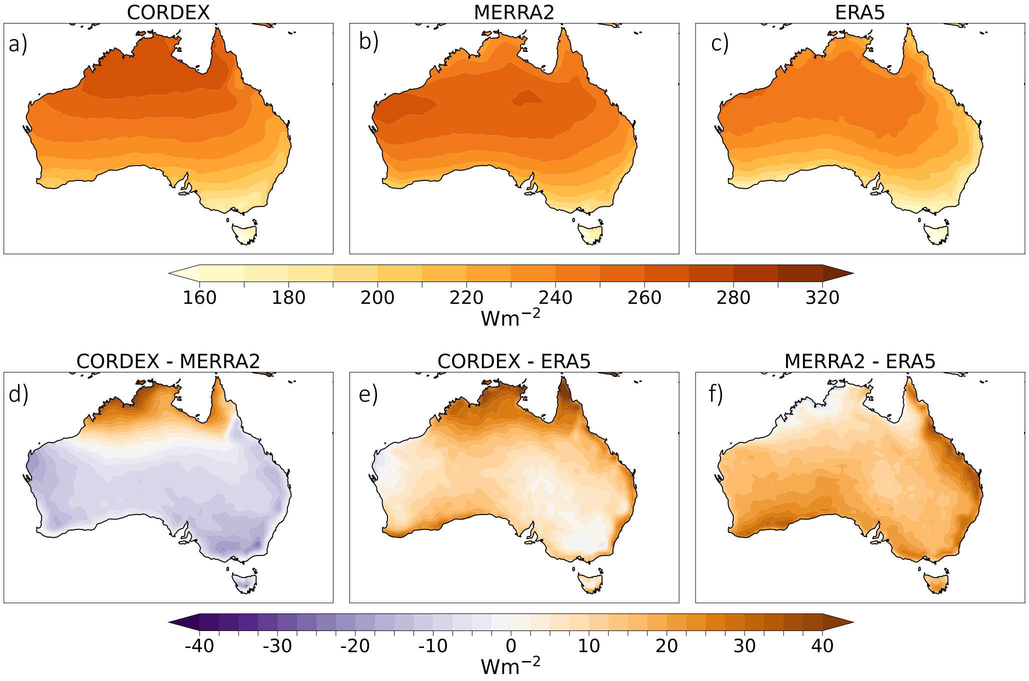

***Figure 1s**. Validation of mean solar radiation of CORDEX-Australasia with two reanalysis products: MERRA2 and ERA5. Panel a-c represents the mean solar radiation from 1980-2005 for a) CORDEX-Australasia ensemble mean, b) MERRA2 c) ERA5. Panel d and e represent the mean bias error ensemble mean of the CORDEX-Australasia simulations when compared with MERRA2 and ERA5, respectively, for the historical period (1980-2005). Panel f) represents the difference in mean downward shortwave radiation in MERRA2 and ERA5 for the historical period (1980-2005).*

In this study, the ensemble mean of shortwave downwelling radiation has been evaluated using two reanalysis products: Modern-Era Retrospective analysis for Research and Applications Version 2 (MERRA2) and European Centre for Medium Range Weather Forecasts (ECMWF) Re-analysis (ERA5), the historical period (1980-2005). We observe a positive bias near Northern Australia in comparison with MERRA2 while negative biases rest of Australia. While there is a similar positive bias in the Northern Australian region in comparison with ERA5 data with negligible positive bias all over the continent. Overall, the CORDEX data captures the solar radiation with reasonable fidelity over Australia, often falling between the two reanalysis. These small differences in the CORDEX-Australasia radiation with the reanalysis products are within an acceptable range. They can be mainly attributed to the sub-grid scale processes

occurring in the actual environment and the specific parameterization schemes selected to obtain the model simulations. This concise evaluation of the surface reaching downwards shortwave radiation in the CORDEX-Australasia ensemble and the previous evaluation studies on CORDEX-Australasia ensembles adds confidence to the results presented in the paper.

2. Details of PV technologies and modelling steps for simulating power with PVLIB

Table 1s. Details of the PV technology used for simulating and validating PV power using PVLIB

	**Mono-Si**	**Multi-Si**	**CdTe**
**Panel Type**	BP-Solar 4170N	Kyocera KD135GX-LP	First Solar FS 272
**Inverter Type**	SMA SMC 6000A	SMA SMC 5000A	Fronius Primo 6.0-1
**Array Structure**	Fixed	Fixed	Fixed
**Array Rating**	5.1 KW	5.4 KW	6.96 KW
**Array Tilt/Azimuth**	20/0	20/0	20/0
**Simulation Period**	2010-2016	2010-2013	2010-2016

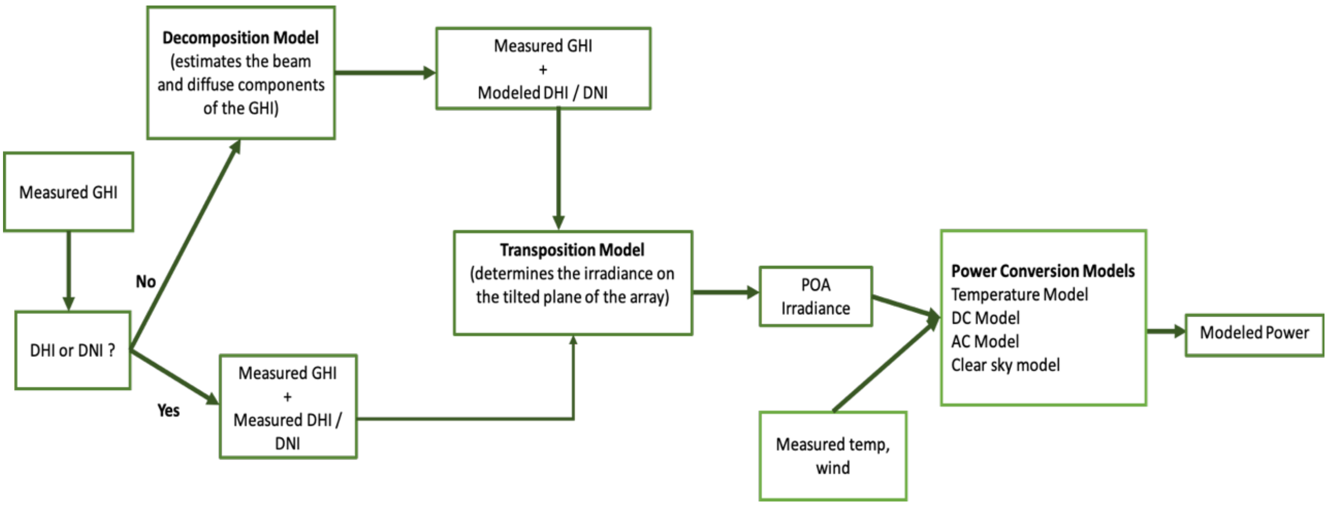

Figure 2s*. Flowchart showing the various modelling steps in PVLIB.*

3. Model Selection for PVLIB power simulations

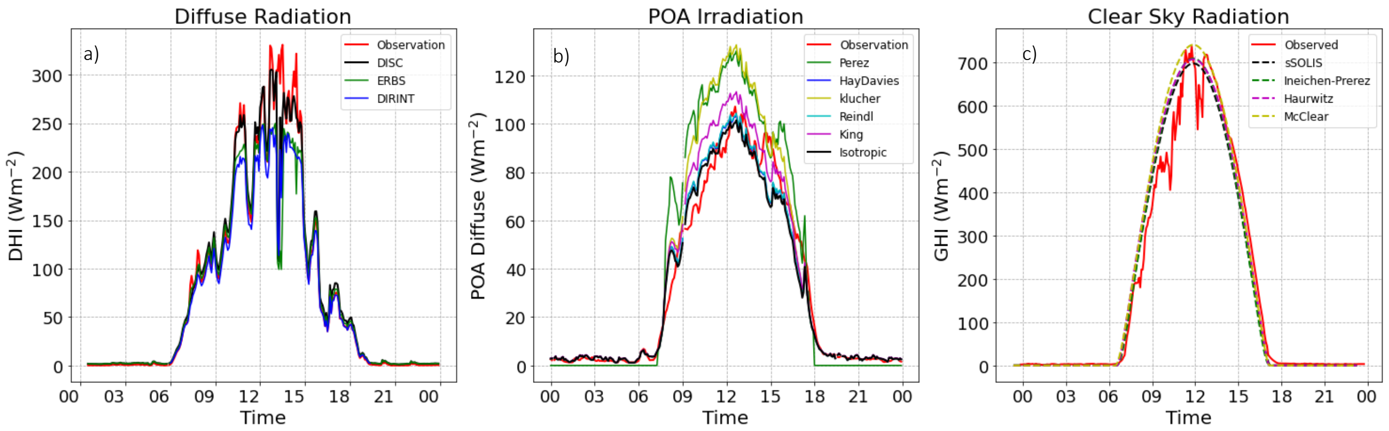

Figure 3s. *Sample DHI, POA diffuse and clear sky radiation at every 5-minute interval from observations and modelled output obtained using PVLIB. Panel a), b) and c) represents the diurnal curves for DHI, POA diffuse irradiation and clear sky irradiation. The red line represents the observations recorded during the day.*

Table 2s. *Error metrics comparing diffuse horizontal irradiance (DHI) obtained from decomposition models with the DHI observations recorded at the weather station at DKASC solar farm*

**Decomposition Models**	**MBE (W/m^2)**	**RMSE (W/m^2)**
**Disc**	10.550885	44.411851
ERBS	12.241542	45.937148
DIRINT	13.694237	52.020097

Table 3s. *Error metrics comparing plane of array (POA) DHI obtained from transposition models with the POA-DHI observations from the weather station at DKASC solar farm.*

**Transposition Models**	**MBE (W/m^2)**	**RMSE (W/m^2)**
**Isotropic**	6.01	43.20
Hay Davies	6.32	44.27
Perez	11.64	47.96
klucher	12.31	46.32
Reindl	6.53	44.35
King	9.517	45.40

Table 4s. *Error metrics comparing clear-sky global horizontal irradiation (GHI) obtained from clear-sky models with the clear-sky GHI from reanalysis product- MERRA2. The modelled GHI is compared with the station observations at DKASC solar farm by extracting GHI during the clear sky periods. The clear-sky periods are calculated following Reno [1].*

**Model**	**MERRA2**		**Observations**	
	**RMSE (W/m^2)**	**Mean Bias (W/m^2)**	**RMSE (W/m^2)**	**Mean Bias (W/m^2)**
**sSOLIS**	23.056	0.914	19.825	-5.229
Inchein-Perez	26.112	-3.916	21.598	-6.172
Haurwitz	31.354	-5.090	28.329	-8.365
McClear	31.15	8.839	22.396	-1.308

4. Validation of PVLIB power simulations with observations from DKASC solar farm

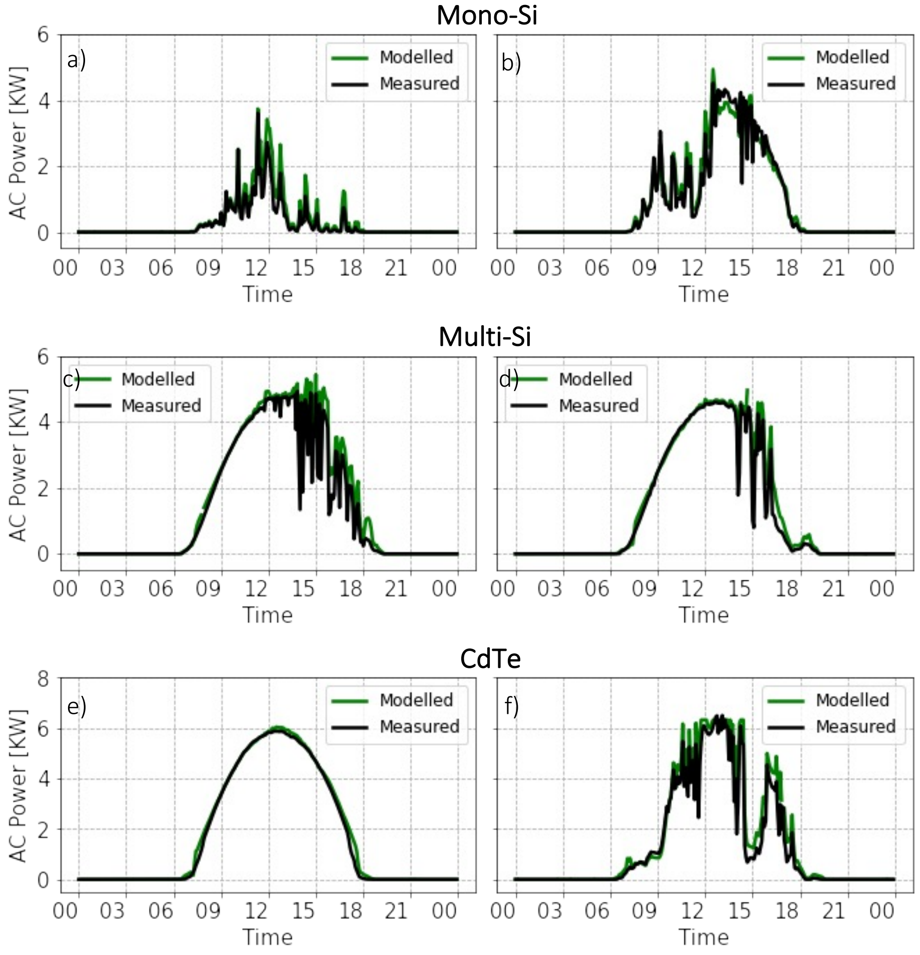

Figure 4s. *Sample PV power output at every 5-minute interval for different PV technologies obtained using PVLIB. Panel a) and b) represent the diurnal power curves for mono-crystalline Silicon cells. Panel c) and d) represent the diurnal power curves for multi-crystalline Silicon cells. Panel e) and f) represent the diurnal curve for thin film Cadmium Telluride cells. The black line represents the AC power output values recorded during the day. The green line represents the modelled AC power using PVLIB.*

Table 5s. *Error metrics comparing power projections obtained from PVLIB simulations and observations recorded at the DKASC solar farm recorded at every 5-minute.*

PV Technology	Mean Bias (KW)	RMSE (KW)
**Mo-Si**	0.02	0.31
**Mu-Si**	0.06	0.39
**CdTe**	0.10	0.54

5. CORDEX-Australasia RCM-GCM pairs used in the study

Table 6s. CORDEX-Australasia GCM-RCM pairs analyzed in this study provided climate projections for both the historical and future periods under RCP8.5 and RCP4.5 scenarios.

RCM / GCM	WRF J	WRF K
**ACCESS1.0**		
**ACCESS1.3**		
**CanESM2**		

6. Mean power projections for Mo-Si cells and future changes for each ensemble member under RCP4.5 and RCP8.5 scenario

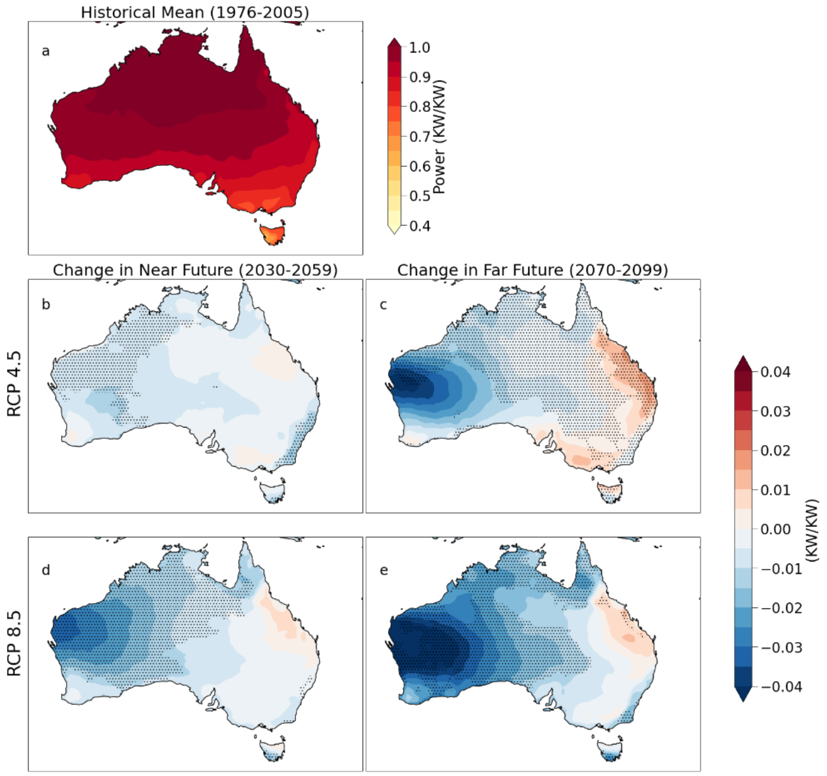

***Figure 5s**. Normalized PV Power across Australia. Panel a) represents the mean normalized power for the historical period (1976-2005). Panel b) and d) represent the future changes in the mean power for the near future (2030-2059) period under RCP4.5 and RCP8.5. Panel c) and e) represents the future changes in the mean power for the far future (2070-2099) period under RCP4.5 and RCP8.5. Stippling indicates a significant change (according to methods: significance test).*

The mean normalized power generation capacity for the monocrystalline Silicon module during the historical period is shown in figure 4s a, and future changes under RCP4.5 and RCP8.5 scenarios are shown in figures 4s b - d for future periods. Due to high solar exposure at those locations, the historical power generation capacity is highest for Northern and Central Australia. During the near future, there is expected to be a slight decline in the power projection throughout the continent, with minor increases in eastern Queensland under both scenarios. There is a significantly higher decrease in the west under RCP8.5 during the near future, unlike RCP4.5. This decline intensifies in magnitude during the far future period for both scenarios. However, it is projected that the East and Southern coastal parts will undergo a small but significant increase in power generation capacity during the far future under RCP4.5, unlike under RCP8.5, where increases are restricted only to Eastern Queensland.

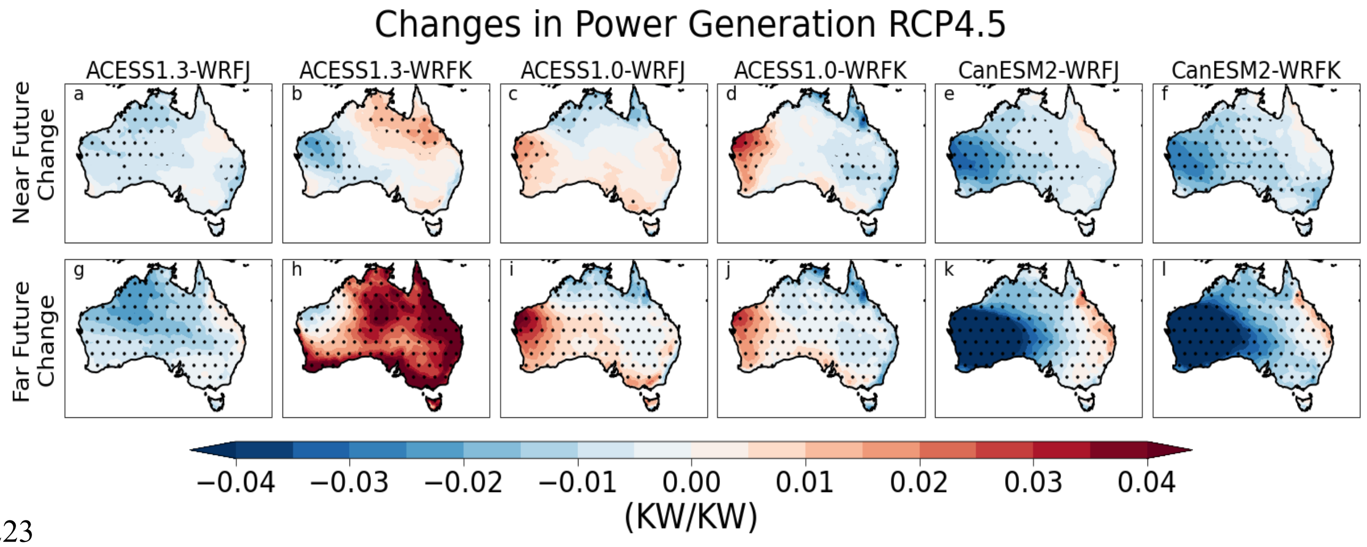

Figure 6s. *Future changes in solar power generation from mono-crystalline silicon cells. Panel a-f represents the future changes in solar power for ensemble members of CORDEX-Australasia for the near future (2030-2059) period under the RCP4.5 scenario. Panel g-l represents the future changes in solar power for ensemble members of CORDEX-Australasia for the far future (2070-2099) period under the RCP4.5 scenario.*

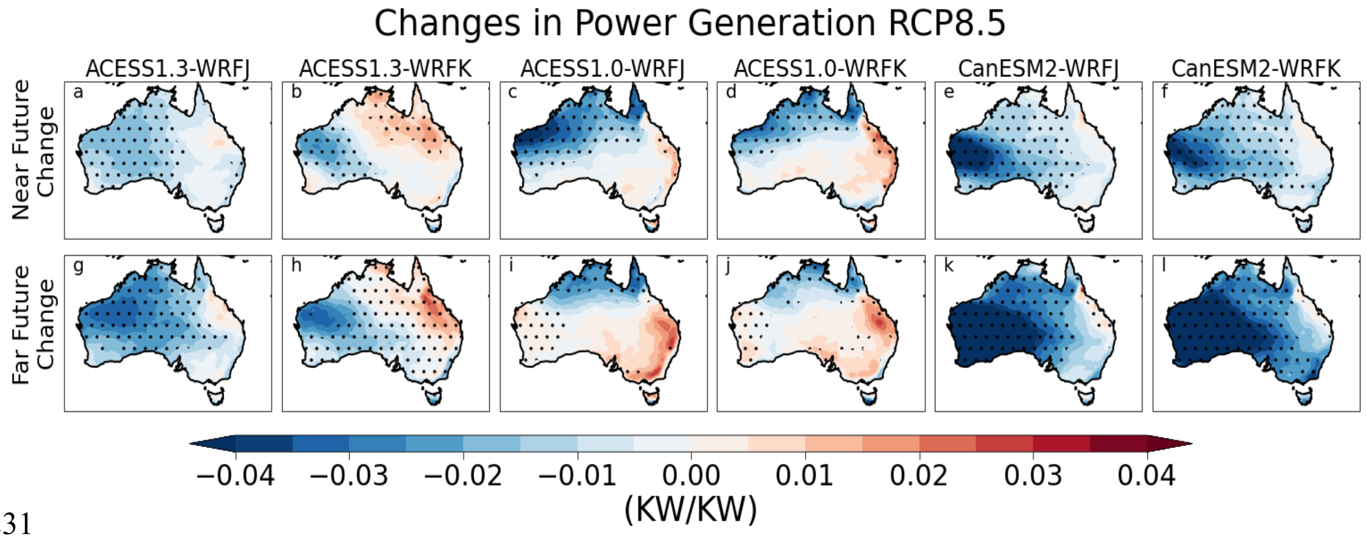

Figure 7s. *Future changes in solar power generation from mono-crystalline silicon cells. Panel a-f represents the future changes in solar power for ensemble members of CORDEX-Australasia for the near future (2030-2059) period under the RCP8.5 scenario. Panel g-l represents the future changes in solar power for ensemble members of CORDEX-Australasia for the far future (2070-2099) period under the RCP8.5 scenario.*

7. Near Future changes for the ramp characteristics across Australia under RCP4.5 and RCP8.5 scenario

Ramp Magnitude:

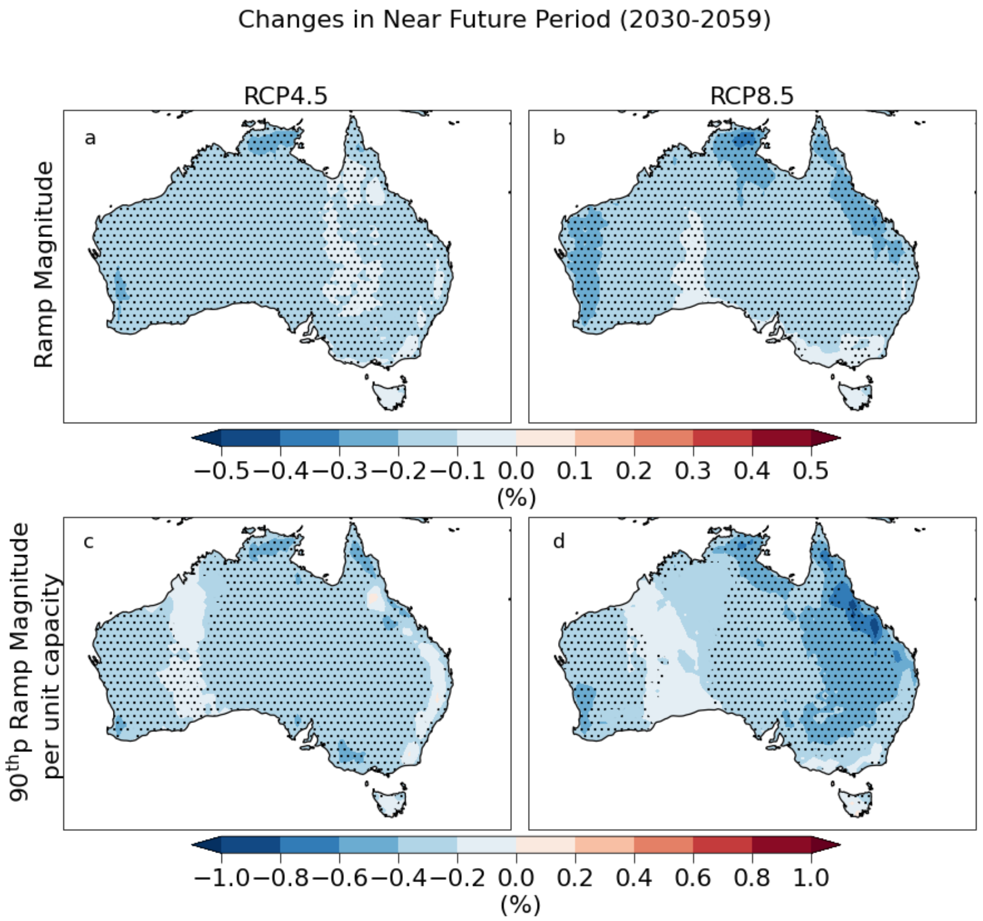

Figure 8s. *Ramp magnitude change across Australia for the near future period (2030-2059). Panel a) and b) represent the future changes in mean ramp magnitude for the far future (2030-2059) period under RCP4.5 and RCP8.5. Panel c) and d) represent the future changes in ramp magnitude at the 90th percentile for the far future (2030-2059) period under RCP4.5 and RCP8.5. Stippling indicates a significant change (according to methods: significance test).*

Ramp Frequency:

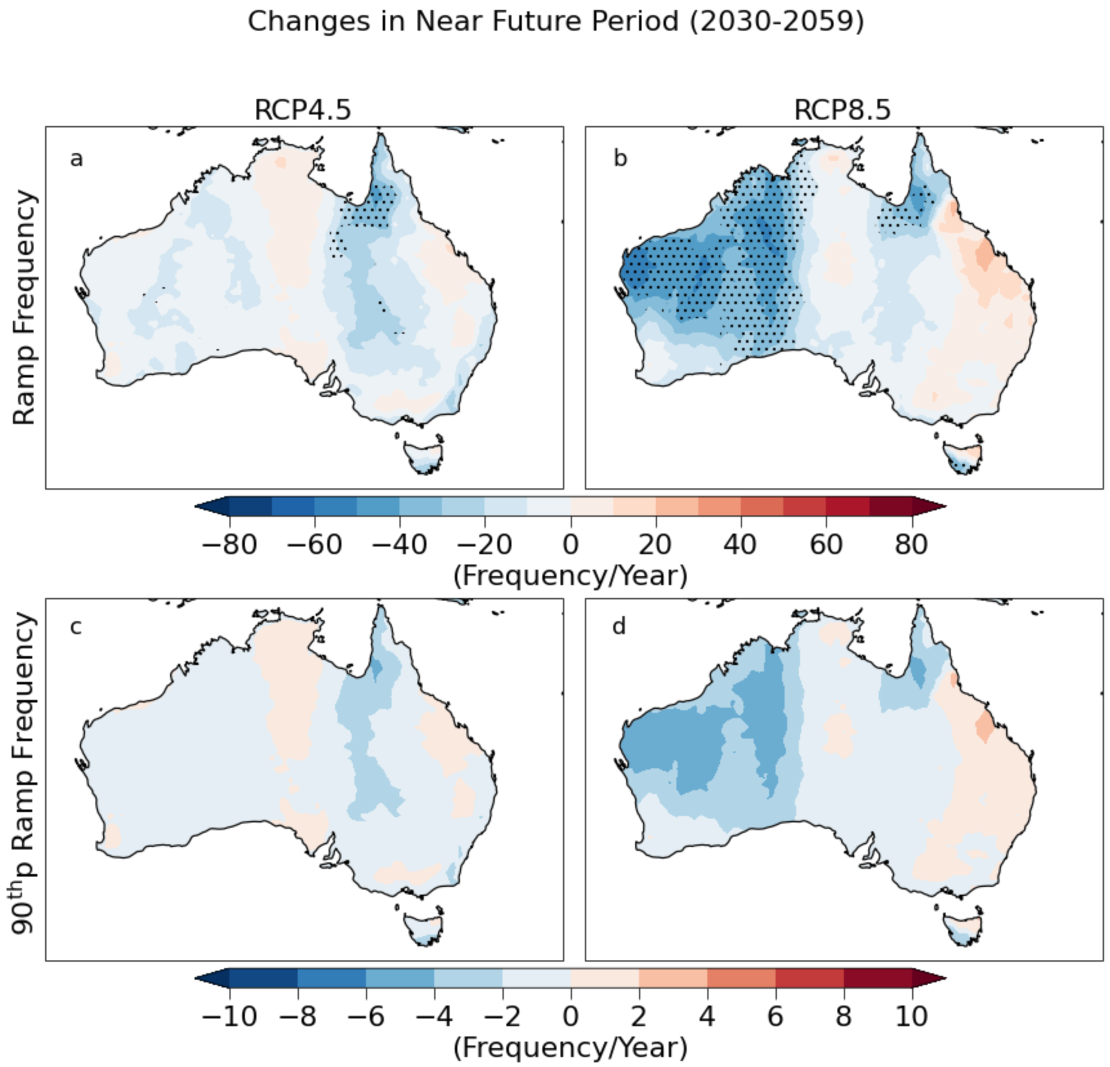

Figure 9s. *Ramp frequency change across Australia for the near future period (2030-2099). Panel a) and b) represent the future changes in mean ramp frequency per year for the near future (2030-2059) period under RCP4.5 and RCP8.5. Panel c) and d) represent the future changes in the frequency of ramps with ramp magnitude at the 90th percentile for the near future (2030-2059) period under RCP4.5 and RCP8.5. Stippling indicates a significant change (according to methods: significance test).*

Ramp Periods:

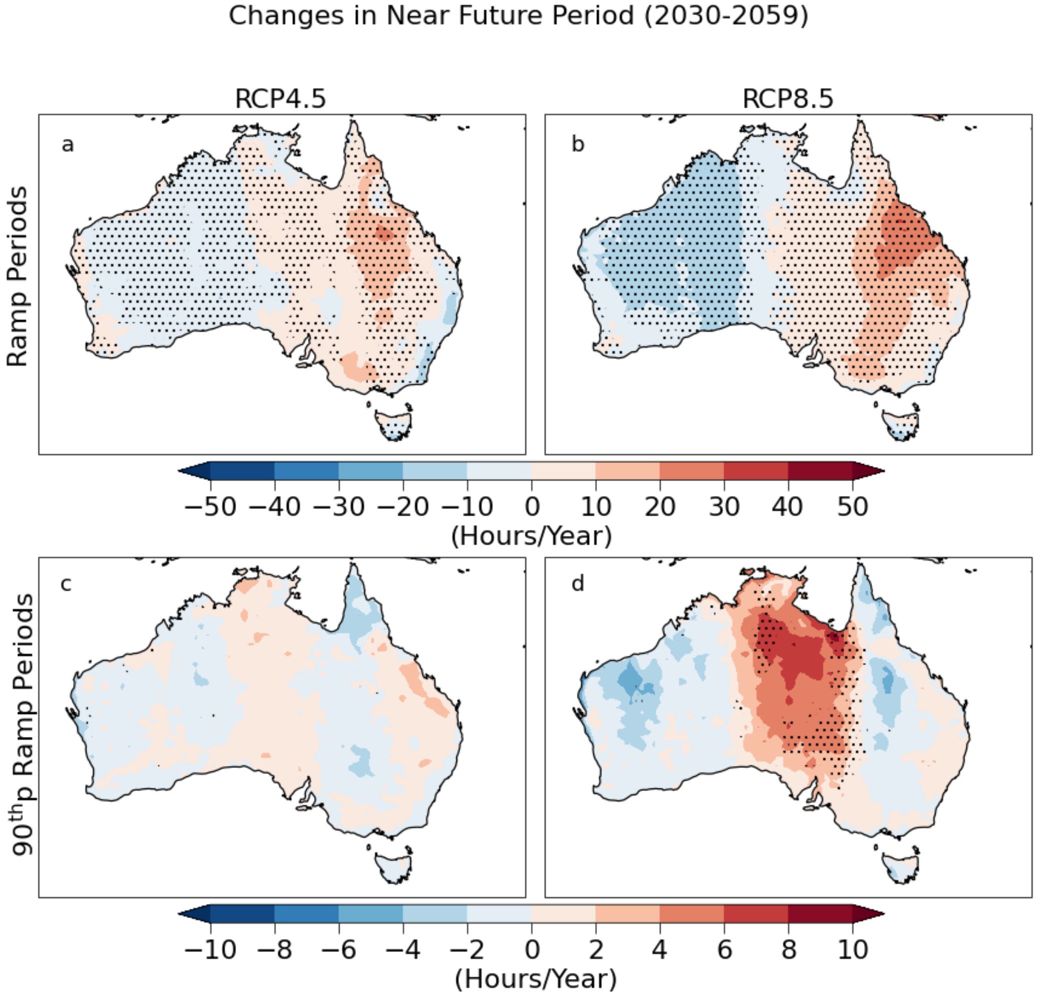

Figure 10s. *Ramp period changes across Australia for the near future period (2030-2059). Panel a) and b) represent the future changes in mean ramp periods per year for the near future (2030-2059) period under RCP4.5 and RCP8.5. Panel c) and d) represent the future changes in ramp periods with ramp magnitude at the 90th percentile for the near future (2030-2059) period under RCP4.5 and RCP8.5. Stippling indicates a significant change (according to methods: significance test).*

8. Future Changes in the mean ramp magnitude for each ensemble member under RCP4.5 and RCP8.5 scenario

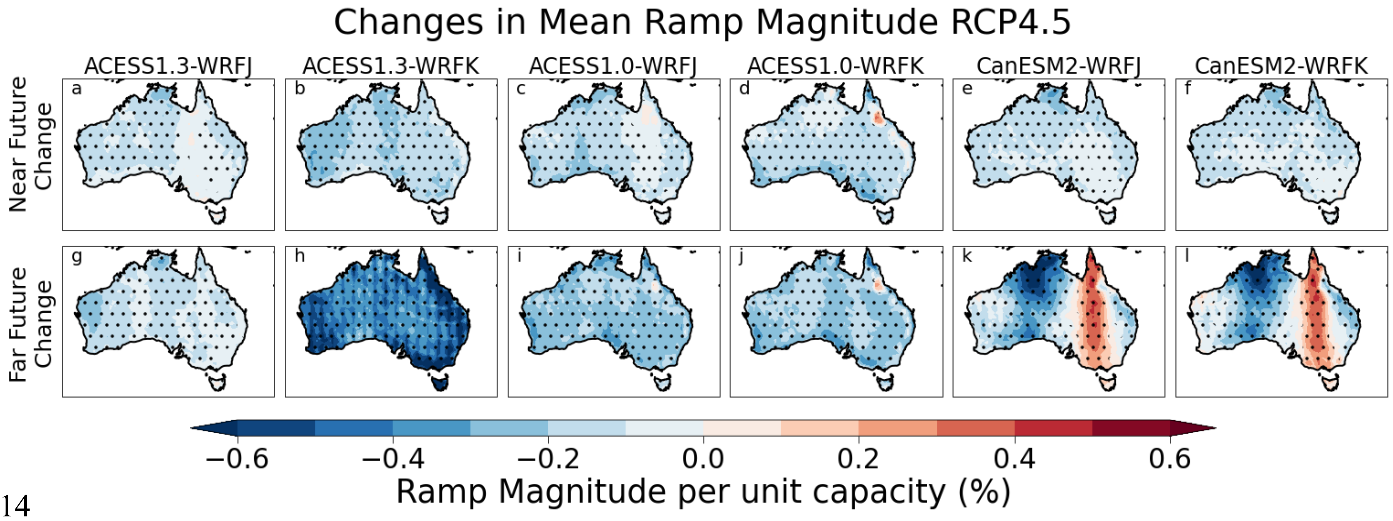

Figure 11s. *Future changes in mean solar power ramp magnitude. Panel a-f represents the future changes in mean ramp magnitude for ensemble members of CORDEX-Australasia for the near future (2030-2059) period under the RCP4.5 scenario. Panel g-l represents the future changes in mean ramp magnitude for ensemble members of CORDEX-Australasia for the far future (2070-2099) period under the RCP4.5 scenario.*

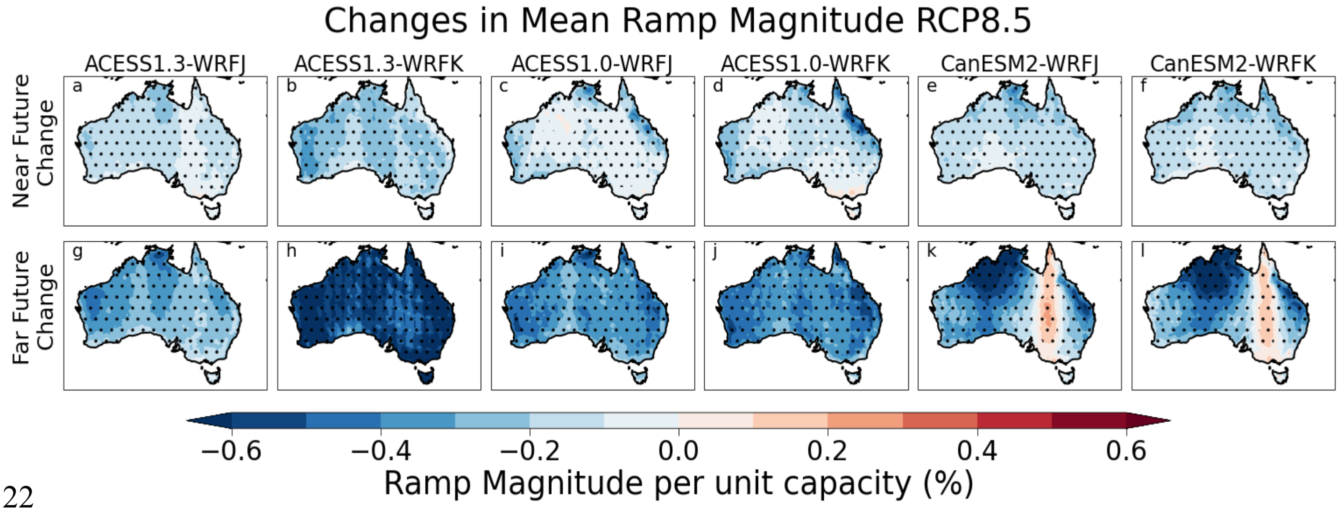

Figure 12s. *Future changes in mean solar power ramp magnitude. Panel a-f represents the future changes in mean ramp magnitude for ensemble members of CORDEX-Australasia for the near future (2030-2059) period under the RCP4.5 scenario. Panel g-l represents the future changes in mean ramp magnitude for ensemble members of CORDEX-Australasia for the far future (2070-2099) period under the RCP4.5 scenario.*

9. Future Changes in the ramp magnitude at 90th percentile for each ensemble member under RCP4.5 and RCP8.5 scenario

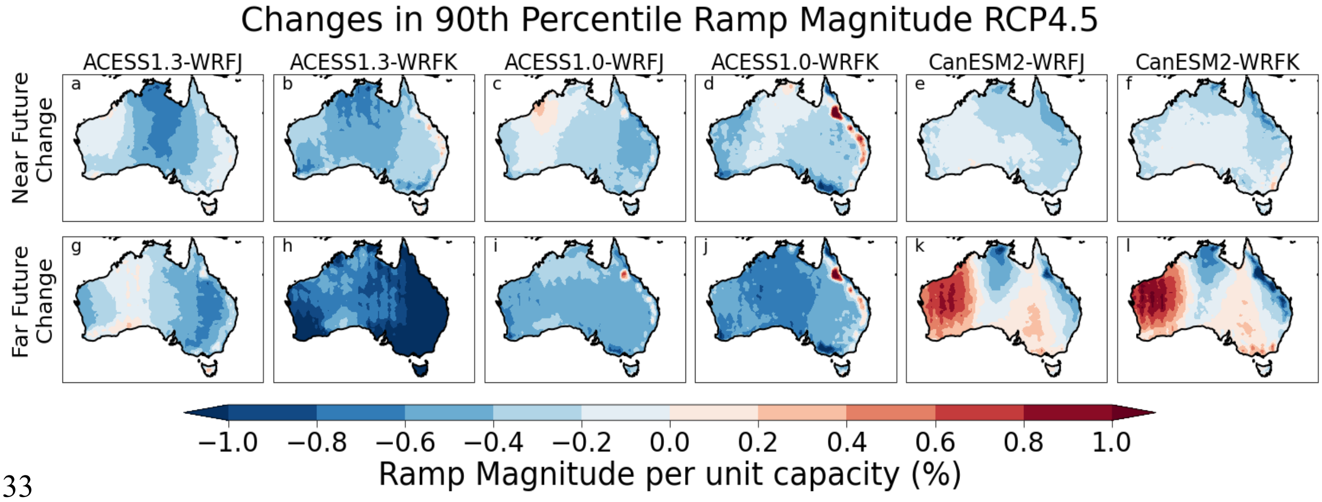

Figure 13s. *Future solar power ramp magnitude changes at the 90th percentile, representing extreme ramps. Panel a-f represents the future changes in extreme ramp magnitude for ensemble members of CORDEX-Australasia for the near future (2030-2059) period under the RCP4.5 scenario. Panel g-l represents the future changes in extreme ramp magnitude for ensemble members of CORDEX-Australasia for the far future (2070-2099) period under the RCP4.5 scenario.*

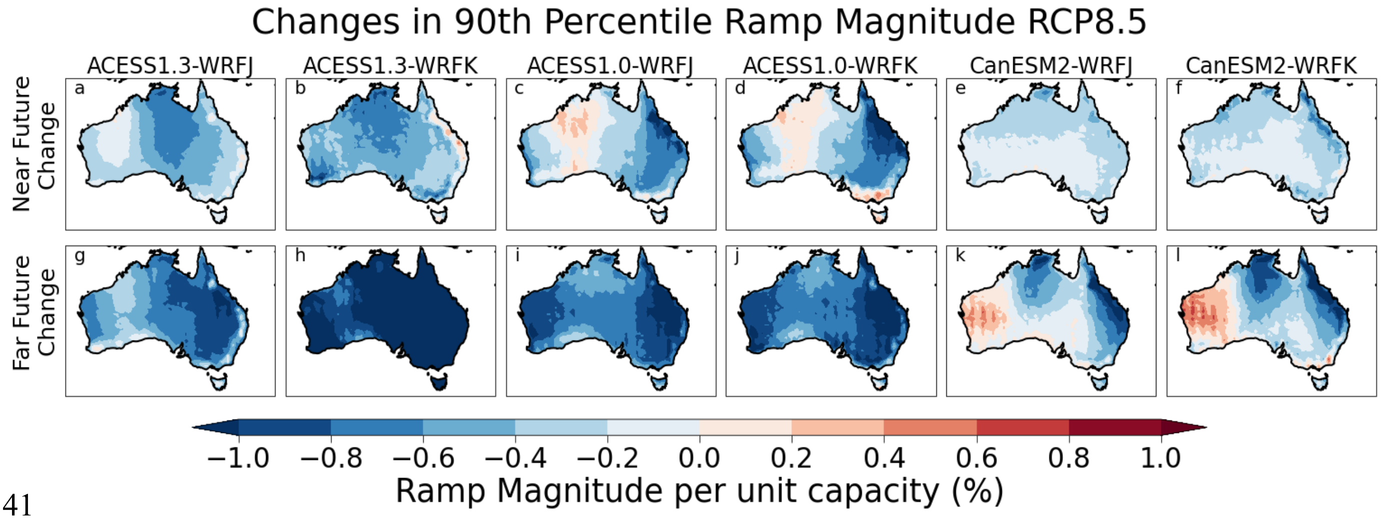

Figure 14s. *Future solar power ramp magnitude changes at the 90th percentile, representing extreme ramps. Panel a-f represents the future changes in extreme ramp magnitude for ensemble members of CORDEX-Australasia for the near future (2030-2059) period under the RCP8.5 scenario. Panel g-l represents the future changes in extreme ramp magnitude for ensemble members of CORDEX-Australasia for the far future (2070-2099) period under the RCP8.5 scenario.*

10. Future Changes in the mean ramp frequency for each ensemble member under RCP4.5 and RCP8.5 scenario

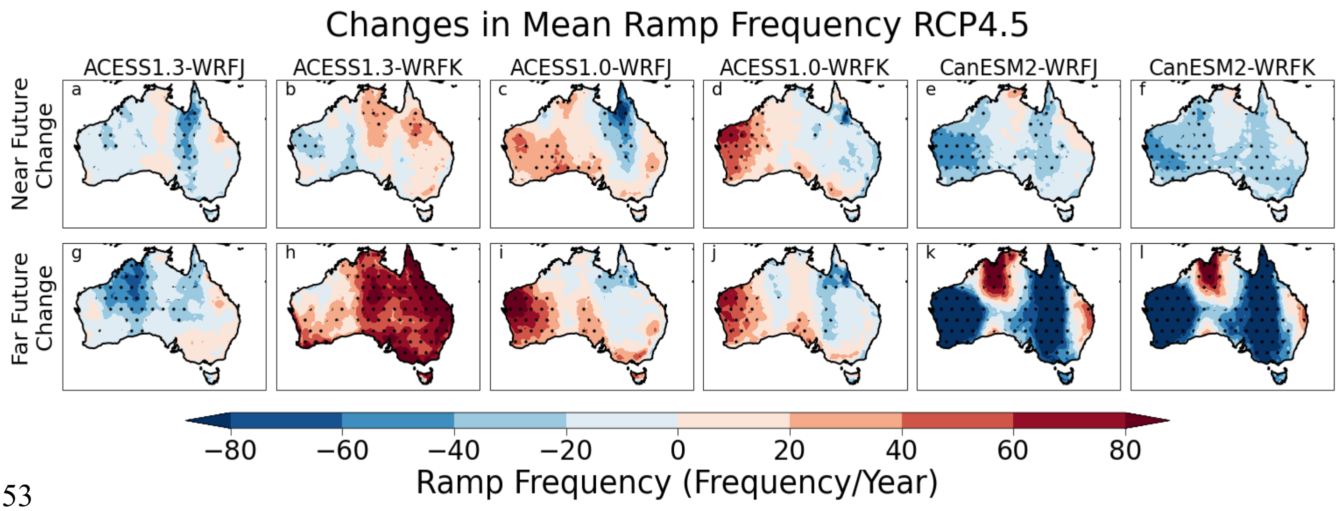

Figure 15s. *Future changes in mean solar power ramp frequency. Panel a-f represents the future changes in extreme ramp frequency for ensemble members of CORDEX-Australasia for the near future (2030-2059) period under the RCP4.5 scenario. Panel g-l represents the future changes in extreme ramp frequency for ensemble members of CORDEX-Australasia for the far future (2070-2099) period under the RCP4.5 scenario.*

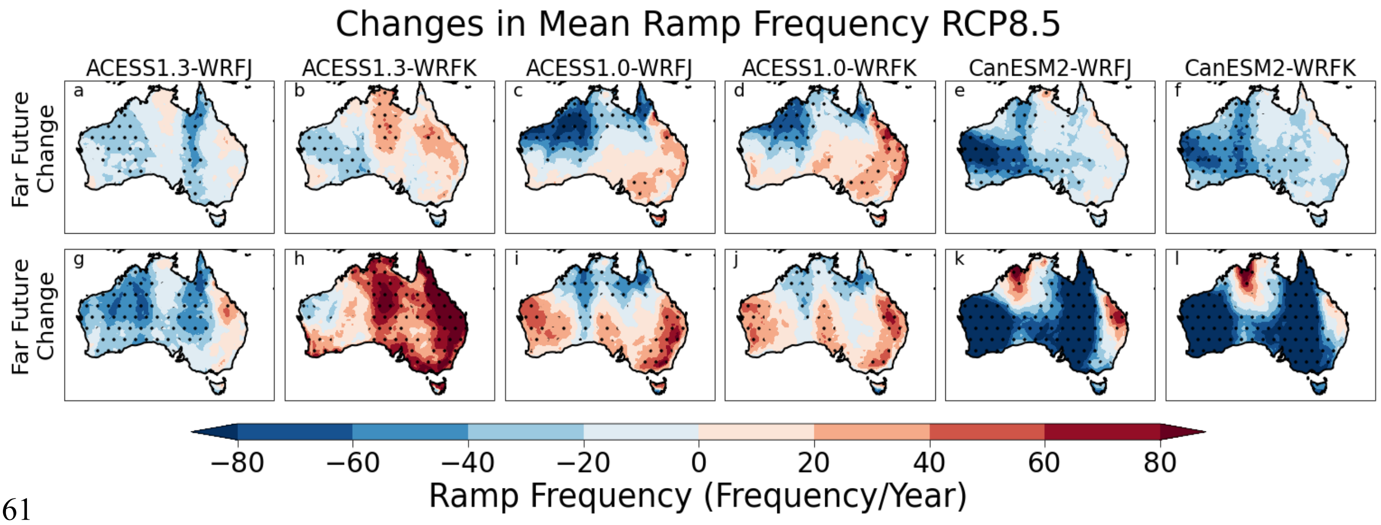

Figure 16s. *Future changes in mean solar power ramp frequency. Panel a-f represents the future changes in extreme ramp frequency for ensemble members of CORDEX-Australasia for the near future (2030-2059) period under the RCP8.5 scenario. Panel g-l represents the future changes in extreme ramp frequency for ensemble members of CORDEX-Australasia for the far future (2070-2099) period under the RCP8.5 scenario.*

11. Future Changes in the frequency of ramp magnitude at the 90th percentile for each ensemble member under the RCP4.5 and RCP8.5 scenario

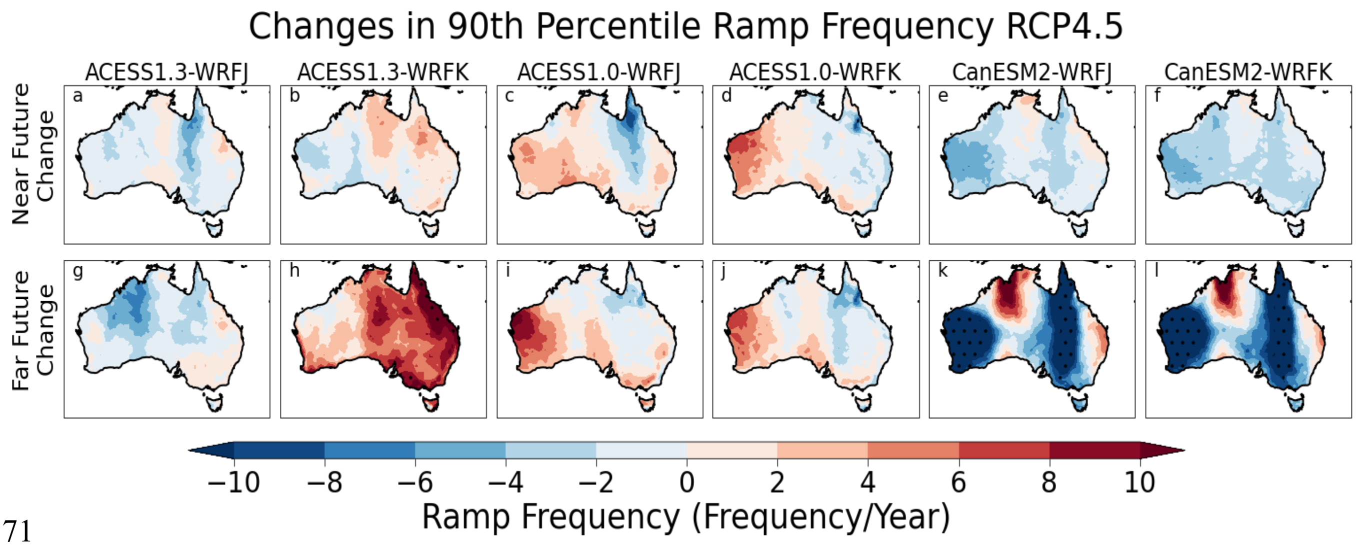

Figure 17s. *Future changes in the frequency of ramps with ramp magnitude at the 90th percentile. Panel a-f represents the future changes in extreme ramp frequency for ensemble members of CORDEX-Australasia for the near future (2030-2059) period under the RCP4.5 scenario. Panel g-l represents the future changes in extreme ramp frequency for ensemble members of CORDEX-Australasia for the far future (2070-2099) period under the RCP4.5 scenario.*

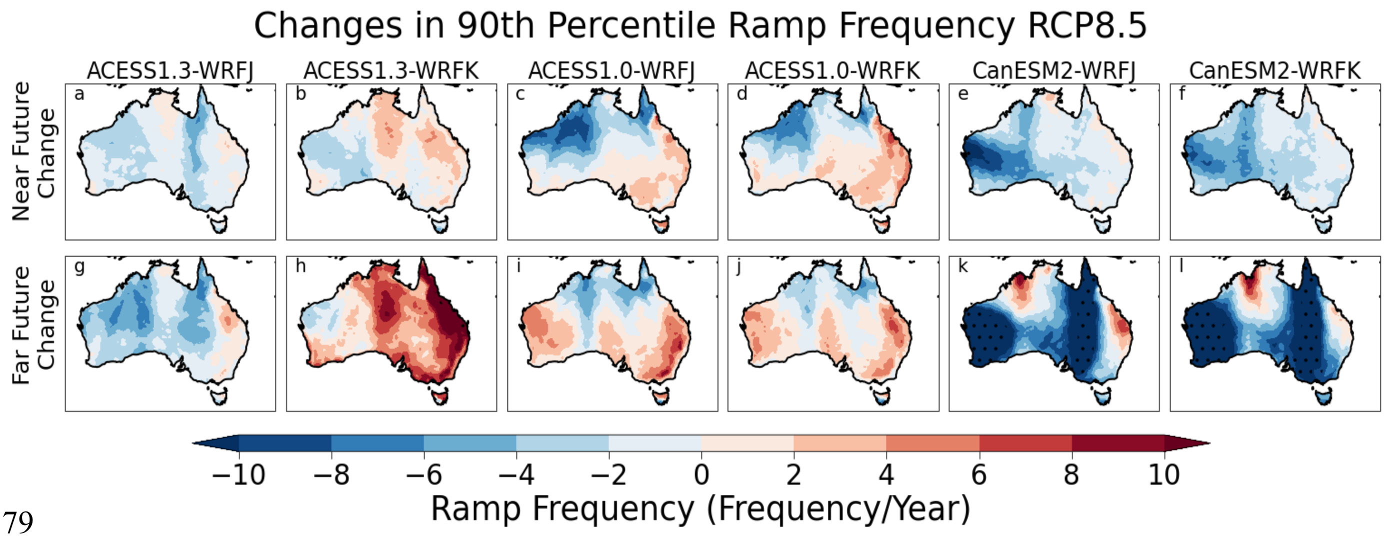

Figure 18s. *Future changes in the frequency of ramps with ramp magnitude at the 90th percentile. Panel a-f represents the future changes in extreme ramp frequency for ensemble members of CORDEX-Australasia for the near future (2030-2059) period under the RCP8.5 scenario. Panel g-l represents the future changes in extreme ramp frequency for ensemble members of CORDEX-Australasia for the far future (2070-2099) period under the RCP8.5 scenario.*

12. Future Changes in the mean ramping periods for each ensemble member under RCP4.5 and RCP8.5 scenario

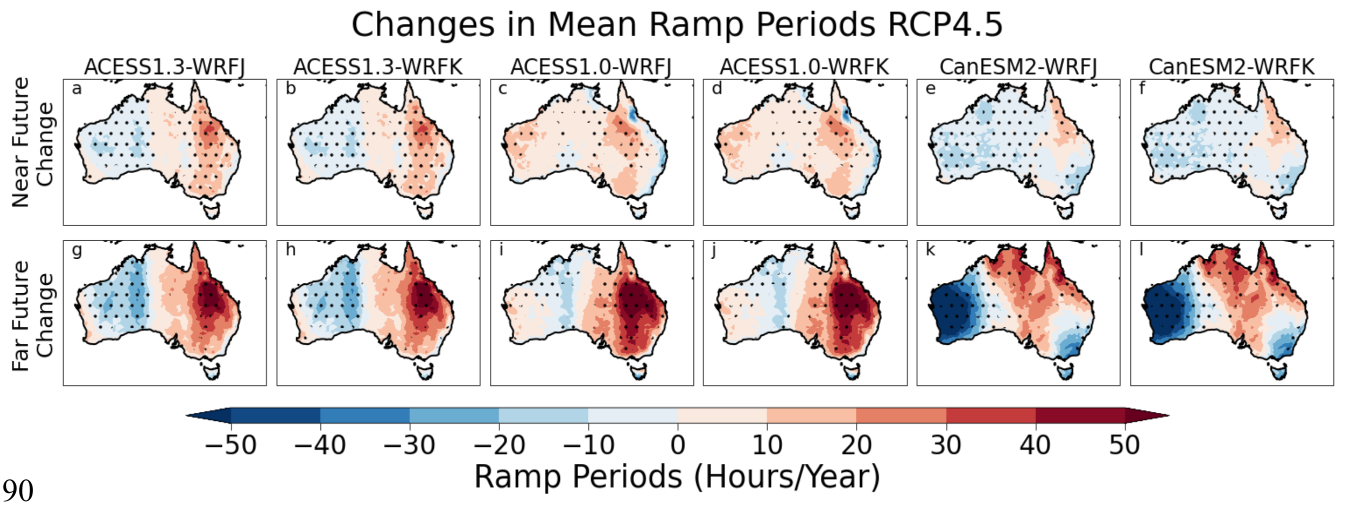

Figure 19s. *Future changes in mean solar power ramping period. Panel a-f represents the future changes in mean ramp periods for ensemble members of CORDEX-Australasia for the near future (2030-2059) period under the RCP4.5 scenario. Panel g-l represents the future changes in the mean ramp period for ensemble members of CORDEX-Australasia for the far future (2070-2099) period under the RCP4.5 scenario.*

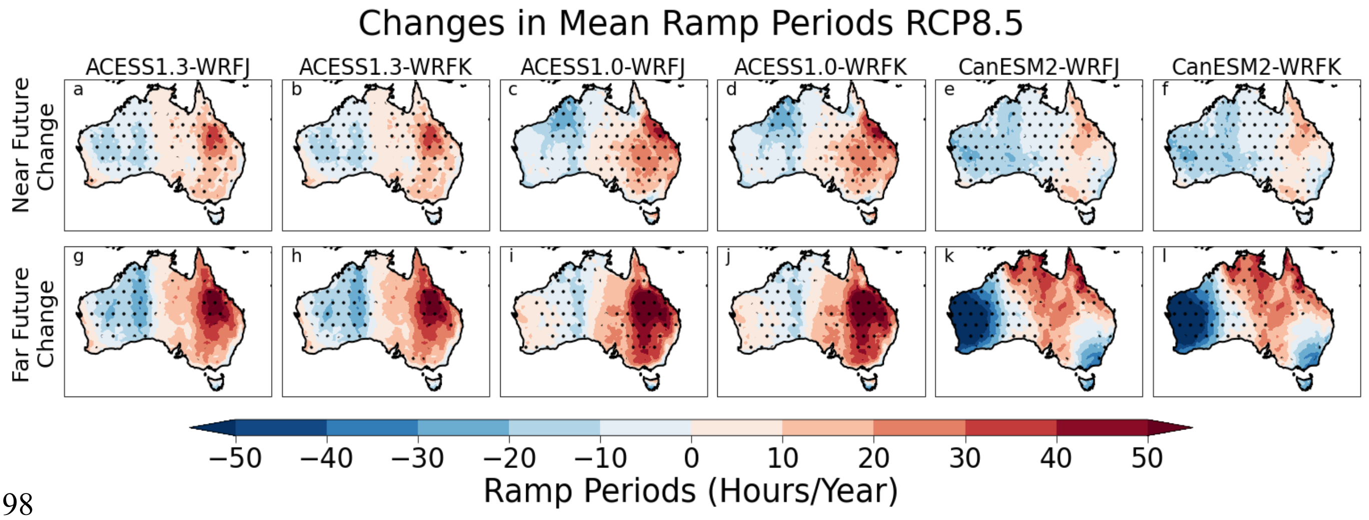

Figure 20s. *Future changes in mean solar power ramping period. Panel a-f represents the future changes in mean ramp periods for ensemble members of CORDEX-Australasia for the near future (2030-2059) period under the RCP8.5 scenario. Panel g-l represents the future changes in the mean ramp period for ensemble members of CORDEX-Australasia for the far future (2070-2099) period under the RCP8.5 scenario.*

13. Future Changes in the ramping period of ramp magnitude at the 90th percentile for each ensemble member under the RCP4.5 and RCP8.5 scenario

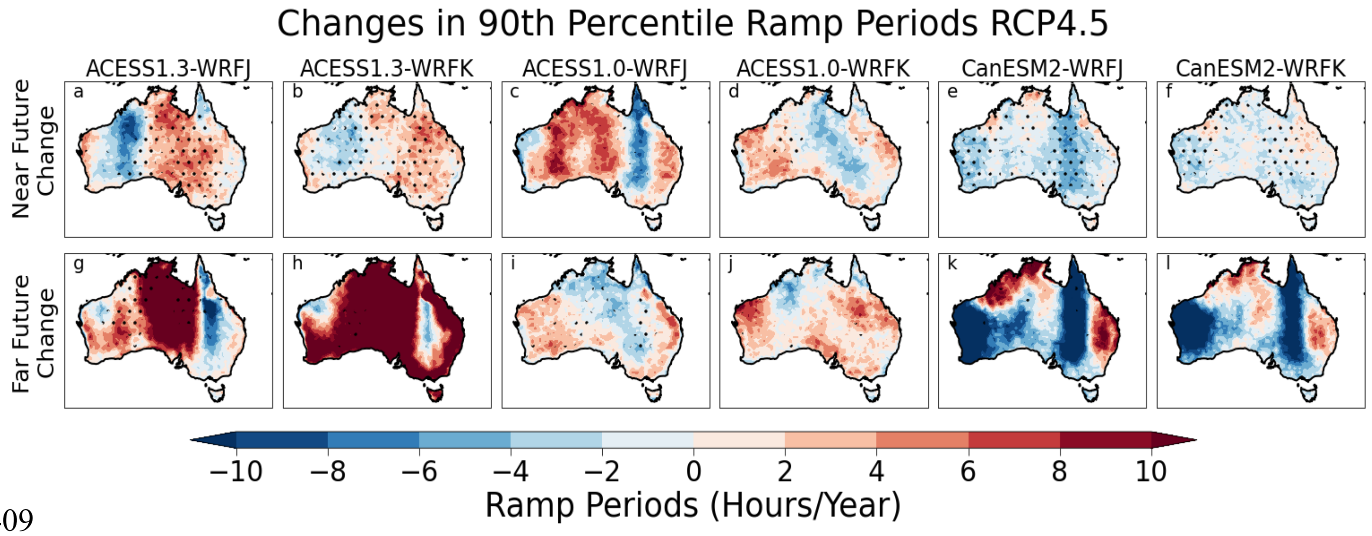

Figure 21s. *Future changes represent the ramp periods with ramp magnitude at the 90th percentile. Panel a-f represents the future changes in the extreme ramp period for ensemble members of CORDEX-Australasia for the near future (2030-2059) period under the RCP4.5 scenario. Panel g-l represents the future changes in the extreme ramp period for ensemble members of CORDEX-Australasia for the far future (2070-2099) period under the RCP4.5 scenario.*

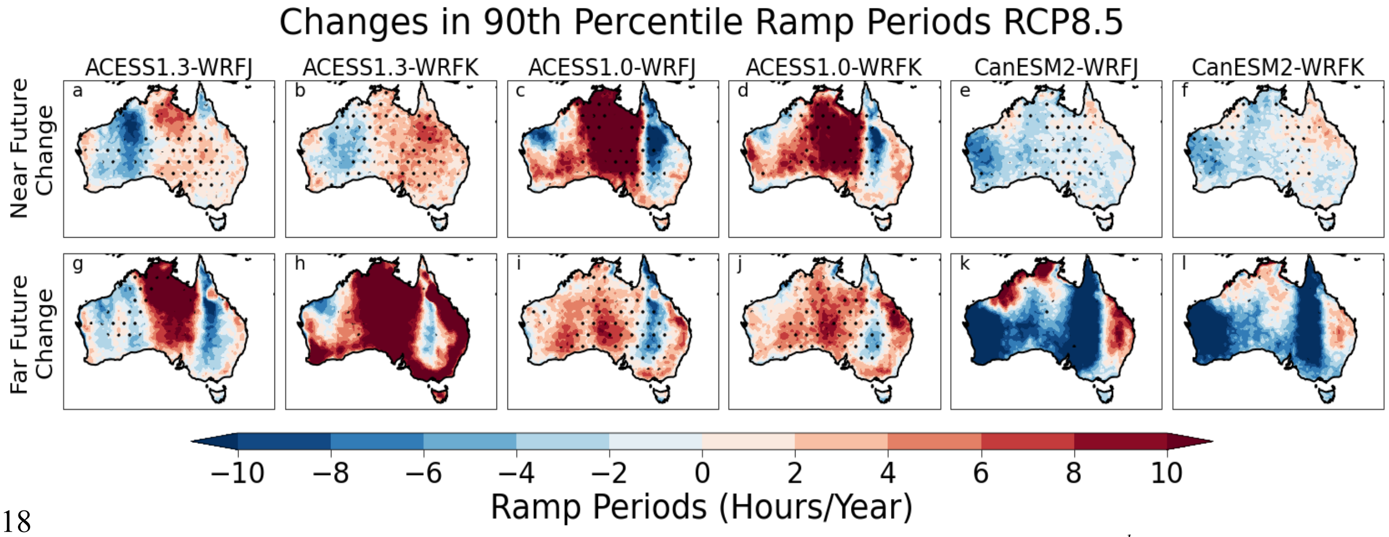

Figure 22s. *Future changes represent the ramp periods with ramp magnitude at the 90th percentile. Panel a-f represents the future changes in the extreme ramp period for ensemble members of CORDEX-Australasia for the near future (2030-2059) period under the RCP8.5 scenario. Panel g-l represents the future changes in the extreme ramp period for ensemble members of CORDEX-Australasia for the far future (2070-2099) period under the RCP8.5 scenario.*

Reference:

[1] Reno MJ, Hansen CW. Identification of periods of clear sky irradiance in time series of GHI measurements. Renew Energy 2016;90:520–31. https://doi.org/10.1016/j.renene.2015.12.031.
